# Development of a coronavirus disease 2019 nonhuman primate model using airborne exposure

**DOI:** 10.1371/journal.pone.0246366

**Published:** 2021-02-02

**Authors:** Sara C. Johnston, Keersten M. Ricks, Alexandra Jay, Jo Lynne Raymond, Franco Rossi, Xiankun Zeng, Jennifer Scruggs, David Dyer, Ondraya Frick, Jeffrey W. Koehler, Paul A. Kuehnert, Tamara L. Clements, Charles J. Shoemaker, Susan R. Coyne, Korey L. Delp, Joshua Moore, Kerry Berrier, Heather Esham, Joshua Shamblin, Willie Sifford, Jimmy Fiallos, Leslie Klosterman, Stephen Stevens, Lauren White, Philip Bowling, Terrence Garcia, Christopher Jensen, Jeanean Ghering, David Nyakiti, Stephanie Bellanca, Brian Kearney, Wendy Giles, Nazira Alli, Fabian Paz, Kristen Akers, Denise Danner, James Barth, Joshua A. Johnson, Matthew Durant, Ruth Kim, Jay W. Hooper, Jeffrey M. Smith, Jeffrey R. Kugelman, Brett F. Beitzel, Kathleen M. Gibson, Margaret L. M. Pitt, Timothy D. Minogue, Aysegul Nalca

**Affiliations:** 1 Virology Division, United States Army Medical Research Institute of Infectious Diseases, Fort Detrick, Frederick, Maryland, United States of America; 2 Diagnostic Systems Division, United States Army Medical Research Institute of Infectious Diseases, Fort Detrick, Frederick, Maryland, United States of America; 3 Veterinary Medicine Division, United States Army Medical Research Institute of Infectious Diseases, Fort Detrick, Frederick, Maryland, United States of America; 4 Pathology Division, United States Army Medical Research Institute of Infectious Diseases, Fort Detrick, Frederick, Maryland, United States of America; 5 Core Laboratory Services Division, United States Army Medical Research Institute of Infectious Diseases, Fort Detrick, Frederick, Maryland, United States of America; 6 Molecular Biology Division, United States Army Medical Research Institute of Infectious Diseases, Fort Detrick, Frederick, Maryland, United States of America; 7 Office of the Science Advisor, United States Army Medical Research Institute of Infectious Diseases, Fort Detrick, Frederick, Maryland, United States of America; 8 Core Support Directorate, United States Army Medical Research Institute of Infectious Diseases, Fort Detrick, Frederick, Maryland, United States of America; Emory University School of Medicine, UNITED STATES

## Abstract

Airborne transmission is predicted to be a prevalent route of human exposure with SARS-CoV-2. Aside from African green monkeys, nonhuman primate models that replicate airborne transmission of SARS-CoV-2 have not been investigated. A comparative evaluation of COVID-19 in African green monkeys, rhesus macaques, and cynomolgus macaques following airborne exposure to SARS-CoV-2 was performed to determine critical disease parameters associated with disease progression, and establish correlations between primate and human COVID-19. Respiratory abnormalities and viral shedding were noted for all animals, indicating successful infection. Cynomolgus macaques developed fever, and thrombocytopenia was measured for African green monkeys and rhesus macaques. Type II pneumocyte hyperplasia and alveolar fibrosis were more frequently observed in lung tissue from cynomolgus macaques and African green monkeys. The data indicate that, in addition to African green monkeys, macaques can be successfully infected by airborne SARS-CoV-2, providing viable macaque natural transmission models for medical countermeasure evaluation.

## Introduction

Severe acute respiratory syndrome coronavirus 2 (SARS-CoV-2), a recently emerged coronavirus, is responsible for an ongoing worldwide pandemic of mild to severe respiratory disease identified as coronavirus disease 2019 (COVID-19). Clinical signs for symptomatic patients have ranged from a mild disease characterized by fever, cough, and/or increased respiratory rate to severe (and sometimes fatal) disease characterized by severe pneumonia, respiratory failure, sepsis/septic shock, and heart failure [1–4]. Asymptomatic infection has also been reported.

Currently there are no licensed vaccines or therapeutics available to prevent or treat SARS-CoV-2. To test and evaluate novel medical countermeasures against SARS-CoV-2, animal models that mimic a natural route of infection and recapitulate human disease are required. To date, countermeasure testing in nonhuman primates has predominantly utilized combination models, wherein high doses of virus in excess of 10^6^ TCID_50_ are inoculated intratracheal (IT)/intranasal (IN) or IT/IN/ocular/oral [5–7]. Although animals in these experiments develop some mild clinical signs of respiratory disease, the routes of exposure are largely artificial in that they rely on direct contact inoculation rather than natural transmission.

Early reports on transmission of SARS-CoV-2 suggested that human spread occurred primarily through respiratory droplets (particles created by coughing and sneezing) and direct contact [2, 8–12]. However, recent evidence indicates that airborne transmission may actually represent the most prevalent human infection route [13]. This route relies on virus-laden particles that are less than 5um in size, which can travel extended distances in the air. Herein, we sought to assess nonhuman primate airborne challenge models of SARS-CoV-2. The data generated was to be used to select the primate species appropriate for further development and countermeasure evaluations. Three nonhuman primate species were evaluated: African green monkeys (AGMs), rhesus macaques (RMs), and cynomolgus macaques (CM). Numerous clinical disease parameters were monitored, including fever, respiratory rate, and lung functionality. Clinical and anatomical pathology were performed to determine potential disease correlates, and qRT-PCR and plaque assay were used to evaluate relative viral load/viremia. Swab samples (oropharyngeal, nasopharyngeal, and rectal) were collected and assayed by qRT-PCR and plaque assay to quantitatively determine viral shedding, and histology and *in situ* hybridization were performed to assess tissue burden and virus-associated pathologic changes. Animals developed mild to moderate respiratory disease, with clinical and hematologic findings consistent with those documented for human disease [4, 14]. In most cases, the changes were more reliably noted and marked for AGMs or CMs. The data indicate that, although all species may be acceptable models for COVID-19, AGMs or CMs may serve as better countermeasure testing models due to the consistency of disease and number of objective parameters that could be used to evaluate efficacy.

High transmissibility and prevalence of this novel coronavirus presents a unique challenge to animal model development efforts. Pre-screening animals for past exposure against most emerging infectious diseases, such as viral hemorrhagic fevers and other lesser-known arthropod borne viruses, often only requires a simple ELISA. In these nonhuman primate (NHP) cohorts, the risk of prior exposure or having cross-reactive antibodies against the challenge agent is low. However, in cases where the risk of prior exposure is high, such as is the case during the SARS-CoV-2 pandemic, a more comprehensive set of assays is required for NHP screening. Herein, we present an integrated assay development approach (PCR, sequencing, immunoassays, and neutralization assays) for SARS-CoV-2 pre-screening of NHP. Through the application of this toolbox, evidence of a previous exposure to SARS-CoV-2 was noted for a colony AGM. Aside from demonstrating the necessity of an integrated screening approach for NHP to be used on COVID-19 studies, it was recognized that this AGM provided a unique opportunity to investigate the effects of prior exposure on disease pathogenesis following re-infection with SARS-CoV-2. Therefore, this animal was exposed to SARS-CoV-2 by the aerosol route alongside the study-enrolled animals. Post-challenge, the same integrated screening approach was applied to longitudinal assessments, and this approach revealed that immunological responses and viral load were significantly impacted by prior exposure to SARS-CoV-2.

## Results

### Clinical disease following SARS-CoV-2 exposure

Healthy, SARS-CoV-2 serologically-naïve African green monkeys (AGM, n = 3), rhesus macaques (RM, n = 4), and cynomolgus macaques (CM, n = 4) were exposed to SARS-CoV-2 on Study Day 1 (i.e. challenge day) by the aerosol route. The mean virus inhaled dose was 3.84x10^4^ plaque forming units (pfu), with AGM receiving an average of 3.80x10^4^ pfu, RM receiving an average of 2.87x10^4^ pfu, and CM receiving an average of 4.86x10^4^ pfu. Daily observations were performed to document clinical signs of disease. None of the animals became terminally ill during the course of the study, and all animals were humanely euthanized at the end of study (Study Day 18 or Study Day 19).

**Fig 1:**
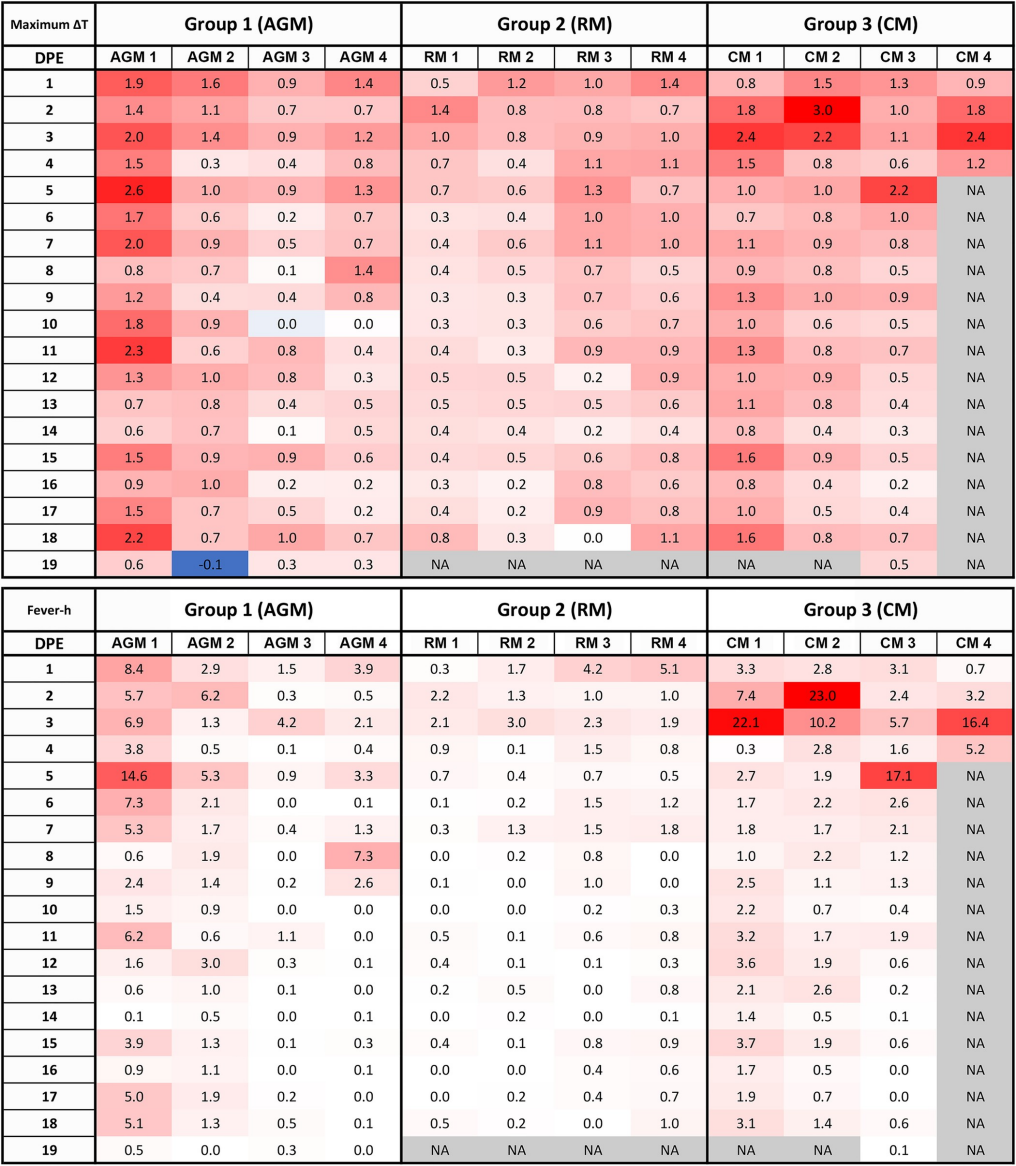
Fever responses as measured by telemetry. DSI M00 telemetry devices were used to collect body temperature data. The figure shows the maximum change in temperature (^o^C) for the 24 hour daily time period.

Clinical observations are described in Table 1. In general, clinical disease findings were noted as early as Study Day 2 and as late as Study Day 18. The earliest clinical sign was the development of fever (defined as a reading that was greater than or equal to 1.5°C above baseline for a duration of greater than 2 hours by telemetry), which was consistently noted for all CM between Study Days 2–3 (Fig 1). The maximum temperature change for CM was between 2.2–3.0°C (mean = 2.5°C). Although small transient temperature increases were noted for some AGM and RM, none met study-defined criteria (greater than or equal to 1.5°C above baseline for a duration of greater than 2 hours) to be called fever. Therefore, fever was a finding exclusive to CM infected with airborne SARS-CoV-2.

**Table 1:** Summary of clinical disease findings.

Animal Number	AGM 1	AGM 2	AGM 3	RM 1	RM 2	RM 3	RM 4	CM 1	CM 2	CM 3	CM 4
Elevated Temp +/- Fever								X	X	X	X
Mild Hypoxia^1^	X	X		X	X	X			X		
Tachycardia		X		X				X	X		X
Increased Respiratory Rate								X	X		
Increased Respiratory Sounds (Auscultation)	X	X	X	X	X	X	X	X	X	X	X
Cough	X										
Lung Infiltrates		X	X	X			X	X	X	X	X
Lung Opacity	X	X	X	X	X	X	X	X	X	X	X
Evidence of Increased Respiratory Effort^2^	X	X	X		X			X	X		
Clotting Abnormality											
Unusual Bruising	X										
Petechial Rash	X										
Erythema of Eyes	X			X		X	X				
Ocular Discharge	X										
Lymphadenopathy			X		X						X
Rectal Bleeding					X	X	X		X	X	
GI Gas/Fluid							X				X
Shaking									X		

^1^Mild hypoxia is defined as 90–94% SpO_2_.

^2^Evidence of increased respiratory effort = abdominal component to breathing and/or nasal flare.

**Fig 2:**
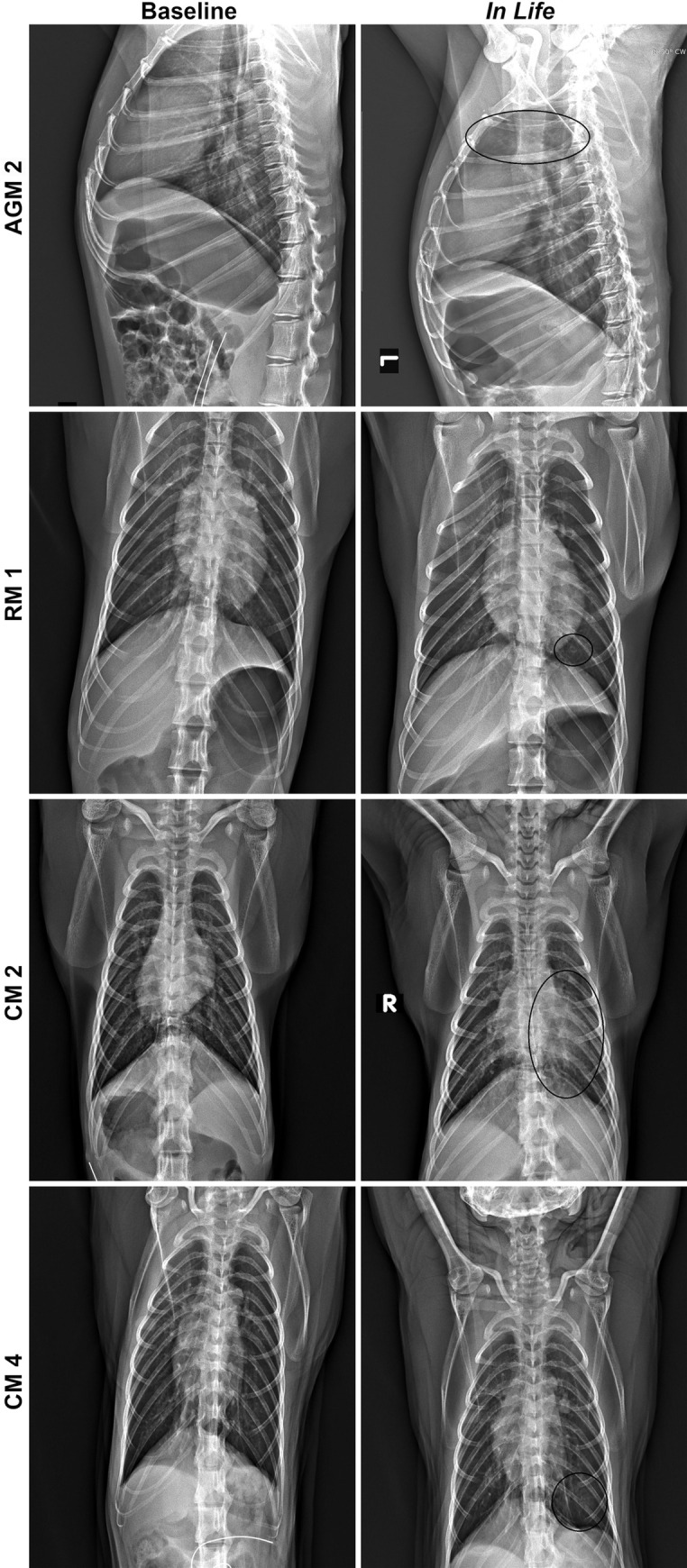
Radiographic changes for SARS-CoV-2 infected NHP. Left panels show baseline images collected prior to telemetry surgery, and right panels show images collected on Study Day 7. All images are ventrodorsal except for AGM 2, which are lateral images. AGM 2 = Infiltrates present in cranial lung lobes (circled). RM 1 = Infiltrate present in caudal left lung (circled). CM 2 = Infiltrate present in left lung (circled). CM 4 = Increased opacity in caudal left lung (circled).

Other common clinical findings for animals on study included increased respiratory sounds on auscultation, and radiographic findings of increased lung opacity with or without the presence of infiltrates (Fig 2). Lung opacity was noted for all animals on study; however, lung infiltrates were more commonly noted for AGM and CM. Generally, radiographic findings were noted prior to audible lung sounds, with radiographic findings commonly identified between Study Days 3–11, and increased respiratory sounds noted between Study Days 5–9.

For AGM, additional findings identified as consistent indicators of disease for this model included mild hypoxia and evidence of increased respiratory effort. For RM, additional consistent findings included mild hypoxia and erythema around the eyes. Rectal bleeding, which was a common finding for this group, was only noted on palpation and likely the result of repeated swabbing leading to irritation and mild bleeding. For CM, an additional consistent finding included tachycardia.

Disease severity based on clinical signs was graded using a scoring system that can be found in S1 Table [Note: this table was based on a table utilized by Finch *et al* [15] and information contained within [16] with certain updates based on the COVID-19 disease observed in the primate species being evaluated in the present study]. Average disease severity scores were as follows (S1 Fig): AGM was 11.00 (range: 8–16), RM was 9.75 (range: 8–11), and CM was 14.00 (range: 10–19). The highest severity score on study was 19 (CM 2), and the lowest severity score on study was 8 (AGM 3 and RM 4).

### Clinical pathology analyses

**Fig 3:**
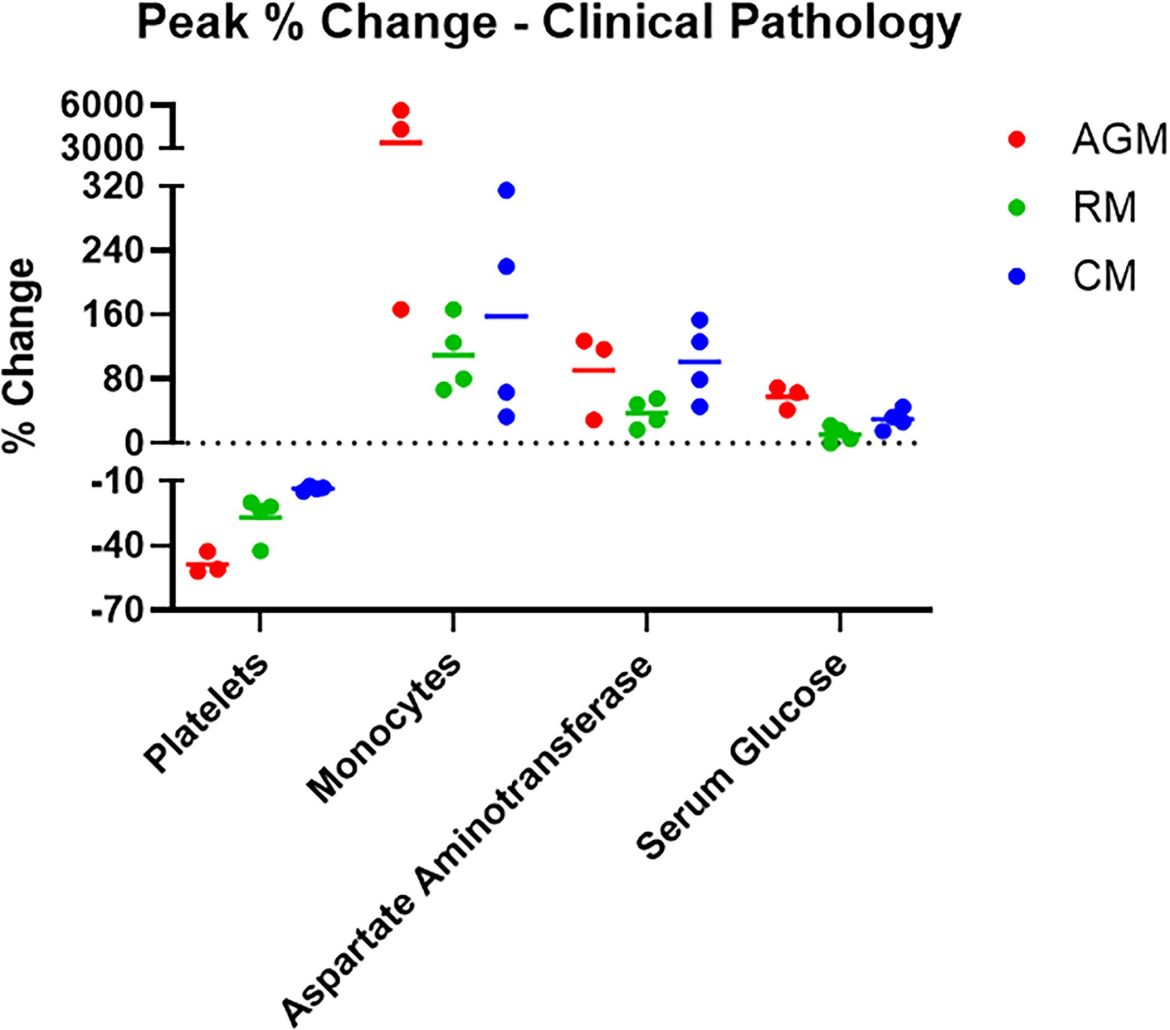
Hematology and clinical chemistries. Hematology was performed on EDTA whole blood using a HM5 instrument. Clinical chemistries were performed on serum using a Piccolo Point-Of-Care instrument. Measurements for animals on study are shown as percent change from baseline (Study Day 1-challenge day values for that animal) for peak values for each analyte.

Complete blood counts and clinical chemistries (Fig 3 and S2 Table) were performed on whole blood and serum, respectively. Peak change from baseline (defined as data from Study Day 1) is represented, and S2 Table depicts the parameters for which changes from baseline were greater than or equal to 25%. Lymphocyte and neutrophil fluctuations were noted for most animals on study and were likely a result of the inflammatory response to infection.

AGM and RM exhibited a decrease in platelet counts beginning on Study Days 3–5 that persisted through Study Days 15–18. Nadirs were reached earlier in AGM (Study Day 5) and were more pronounced (40%) compared to RM. Decreases may relate to lung injury and subsequent sequestration; evidence of inflammation and chronic lesions noted histologically further support this assessment.

Increases in one or more liver-related enzyme activities were present for most animals, with the most notable and consistent changes occurring for AGMs and CMs. For CMs, elevated levels appeared to correlate with the timing of the fever response, with levels approximating baseline by Study Day 7. A more protracted response was noted for AGMs, with elevated levels noted between Study Days 5–11. Unfortunately, necropsy occurred 7–11 days after enzyme activities had returned to baseline, so any corresponding histologic changes in the liver that might have suggested a cause for the elevations were no longer present.

Increases in glucose concentration were noted in AGM over Study Days 3–7 (1.3 fold) and 15–18 (1.6 fold). A similar trend was noted for CMs, but the degree and consistency of the change was less pronounced. These findings may relate to a corticosteroid or epinephrine response. Changes in glucose levels were not noted for RMs.

Significant increases in monocytes, measured on Study Day 5, were present only for AGM; a 57 fold and 44 fold increase from baseline was noted for AGM 2 and AGM 3, respectively. Although the physiological relevance of this finding is unclear at this time, monocytosis was a consistent and exclusive finding for AGM. No other notable changes in clinical chemistry parameters were measured on this study.

### Viral shedding

**Fig 4:**
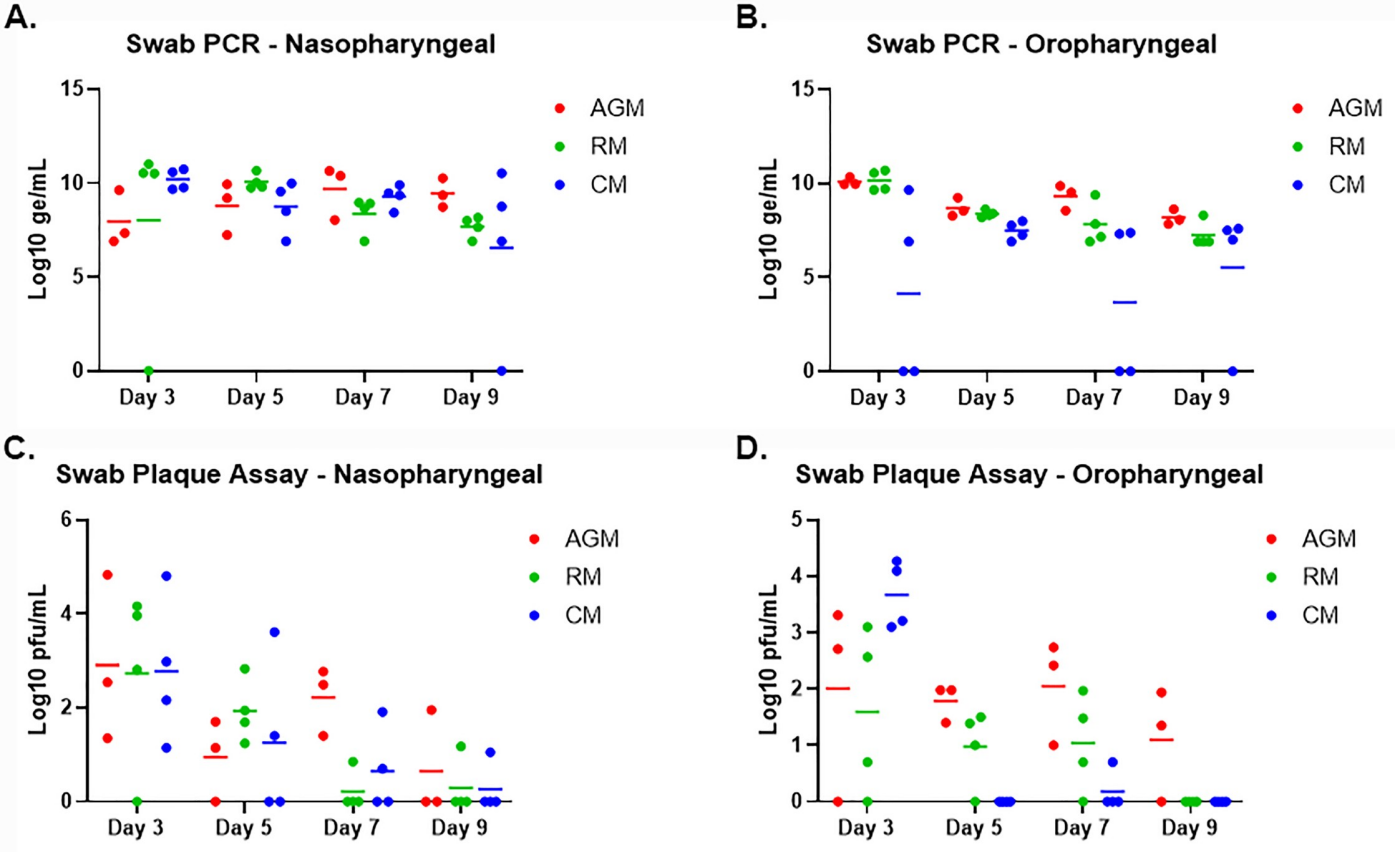
Viral RNA and live virus in oropharyngeal and nasopharyngeal swabs. SARS-CoV-2-specific qRT-PCR (A-B) was performed on RNA extracted from nasopharyngeal and oropharyngeal swab clarified homogenates. Plaque assay (C-D) was performed on nasopharyngeal and oropharyngeal clarified homogenates. Data are shown as Log_10_ ge/mL (qRT-PCR) or Log_10_ pfu/mL (plaque assay), with nasopharyngeal swab data for each species shown in A and C, and oropharyngeal swab data for each species shown in B and D.

Quantitative reverse transcription polymerase chain reaction (qRT-PCR) was conducted on oropharyngeal (OP) and nasopharyngeal (NP), and rectal swabs to assess viral shedding (Fig 4 and S2 Fig). Viral RNA in rectal swabs, when noted, was measured on or after Study Day 7; this finding was noted for two AGM and two RM, was either noted on Study Day 7 or Study Day 15, and did not correlate with the presence of infectious virus as determined by plaque assay. Viral RNA was detected for most animals by Study Day 3 in OP and NP swabs. Peak levels in NP swabs were between 9.63–11.02 Log_10_ genomic equivalents (ge)/mL, and peak levels in OP swabs were between 7.50–10.70 Log_10_ ge/mL. Significant differences in peak levels between swab types and groups were not observed with one exception: CM. For this group, peak levels in OP swabs were 1–2 logs lower compared to OP swab peak levels for other groups. For the majority of the animals, viral RNA was no longer detected by Study Day 11. Viral RNA present for a few AGM on Study Day 18 likely represented residual RNA in the nasal cavity and not actively replicating virus.

Viability of virus in OP and NP swabs was confirmed by plaque assay (Fig 4); viable virus was not detected in any rectal swabs. Virus was detected for most animals by Study Day 3 in both swab types, with peak levels typically detected on Study Day 3. Peak levels in NP swabs were between 2.16–4.83 Log10 pfu/mL, and peak levels in OP swabs were between 2.71–4.45 Log10 pfu/mL; significant differences between swab types and groups were not observed. For the majority of the animals, virus was no longer detected by Study Day 9; however, virus was still detected on Study Day 9 and/or 11 for a few animals (AGM 1 OP and NP, AGM 2 OP, RM 2 NP, RM 3 NP, and CM 3 NP).

### Gross, histological and molecular pathology

Gross necropsy findings were infrequent and when they were observed, the majority of lesions were confined to the lungs. The most common finding was discoloration of one or more lung lobes for AGM and CM. In two AGM (AGMs 1 and 2), diffuse red mottling of all lobes was noted, and in CM 3 locally extensive red discoloration of all lobes was observed. CM 2 also had multifocal red to gray discoloration of the right caudal lung lobe. Two animals had adhesions within the lungs and/or thorax cavity. AGM 2 had thin, clear adhesions that extended from the left cranial to the left caudal lobe, with similar adhesions noted on the right side that involved all four lobes giving the appearance of a large, single lobe on each side. CM 4 had multiple, thin, clear adhesions that extended from the right middle lobe to the pleura of the thoracic wall.

No viral RNA was detected in any animal by *in situ* hybridization, indicating viral infection had been cleared in these tissues at the time when animals were euthanized at the end of study.

**Fig 5:**
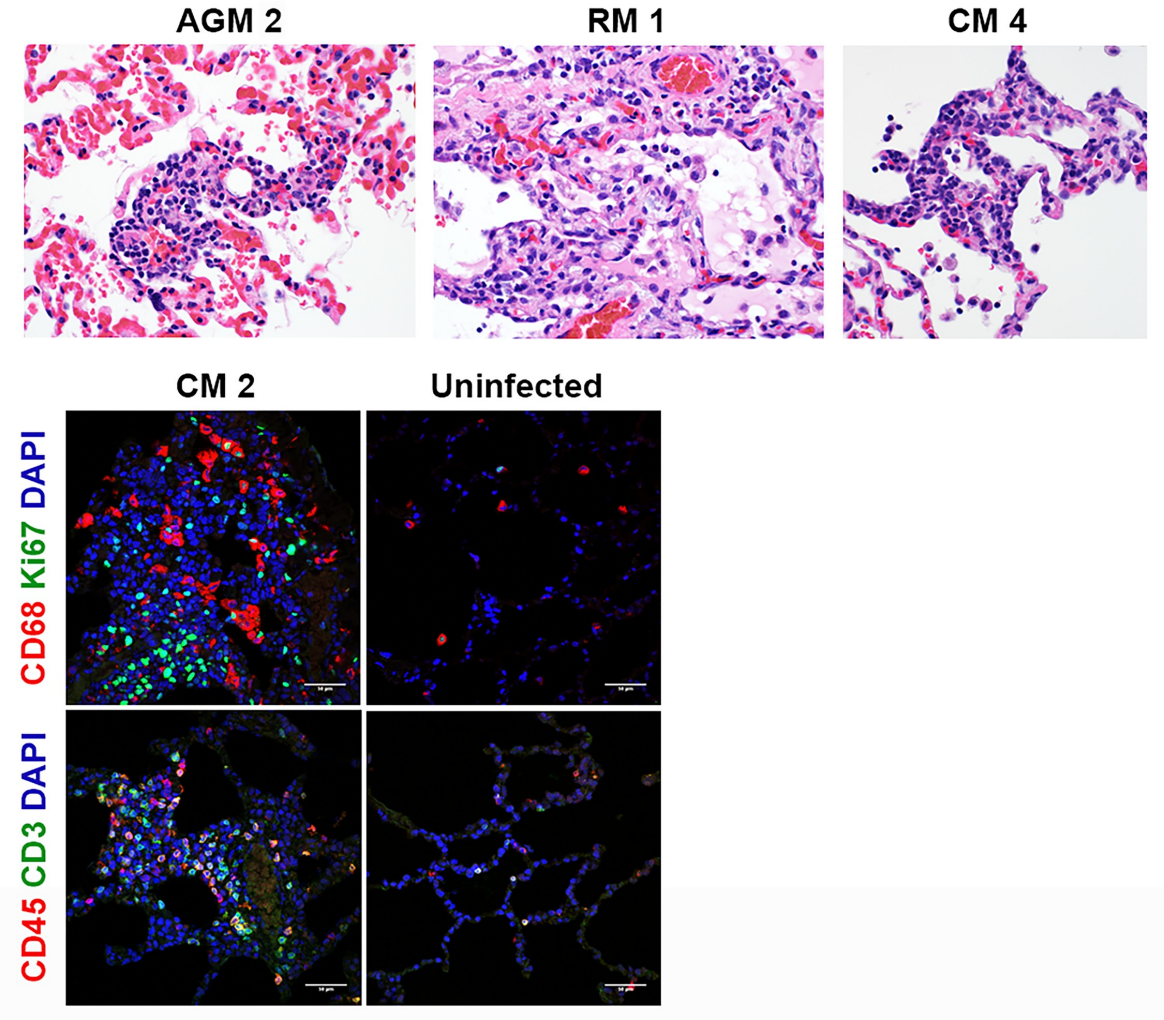
Histopathology and IFA. Necropsies, histopathology, and IFA were performed on all animals. The top panels show the following histopathology findings: AGM 2 and CM 4 = type II pneumocyte hyperplasia; RM 1 = type II pneumocyte hyperplasia and alveolar fibrosis. The bottom panels show excessive CD68^+^ macrophages (red) and Ki67^+^ proliferating cells (green), CD45^+^ leukocytes (red), and CD3^+^ T cells infiltrated in alveolar septum for CM 2 compared to uninfected control lung tissue.

Representative images for histology are shown in Fig 5. With the exception of CM 1, all animals had some degree of inflammation and chronic lesions (alveolar fibrosis, type II pneumocyte hyperplasia) in one or more lung lobes. The alveolar fibrosis and type II pneumocyte hyperplasia indicate previous cellular damage, either due to the viral infection and/or resulting inflammation, and these findings were more frequently noted for AGM and CM. Multinucleate cells likely attributed to viral infection were only noted for AGMs 2 and 3. Multinucleate cells possibly related to viral infection were also found in the axillary lymph node for AGM 3 and the inguinal lymph node for CM 3. Other histologic findings included the following: inflammation in the nasal septum/turbinates for AGM 2, RM 1, and CMs 2 and 3; lymphoid hyperplasia in lymph nodes, mucosa-associated lymphoid tissue, and/or gut-associated lymphoid tissue nearly all animals.

Representative images for IFA are shown in Fig 5. Consistently, in comparison to historical uninfected cynomolgus macaque lung tissues, infiltration of inflammatory cells including CD68^+^ macrophages, Ki67^+^ proliferating cells, CD45^+^ leukocytes, and CD3^+^ T cells were multi-focally detected in alveolar septum of CM 2.

### Immunoassay development

We took a multi-pronged assay development/implementation approach by producing in-house assays, and utilizing these in conjunction with a commercial, EUA approved ELISA kit (Euroimmun SARS-CoV-2 S1 IgG ELISA kit). In-house assays were optimized in conjunction with study pre-screening, utilizing available pre-challenge study samples (serum and NP swabs) for this effort. These assays included a bead-based immunoassay on the Magpix platform for detection of SARS-CoV-2 IgM and IgG responses, and a plaque reduction neutralization test (PRNT) for the measurement of neutralizing antibody titers to SARS-CoV-2.

### SARS-CoV-2 pre-exposure in NHP

**Fig 6:**
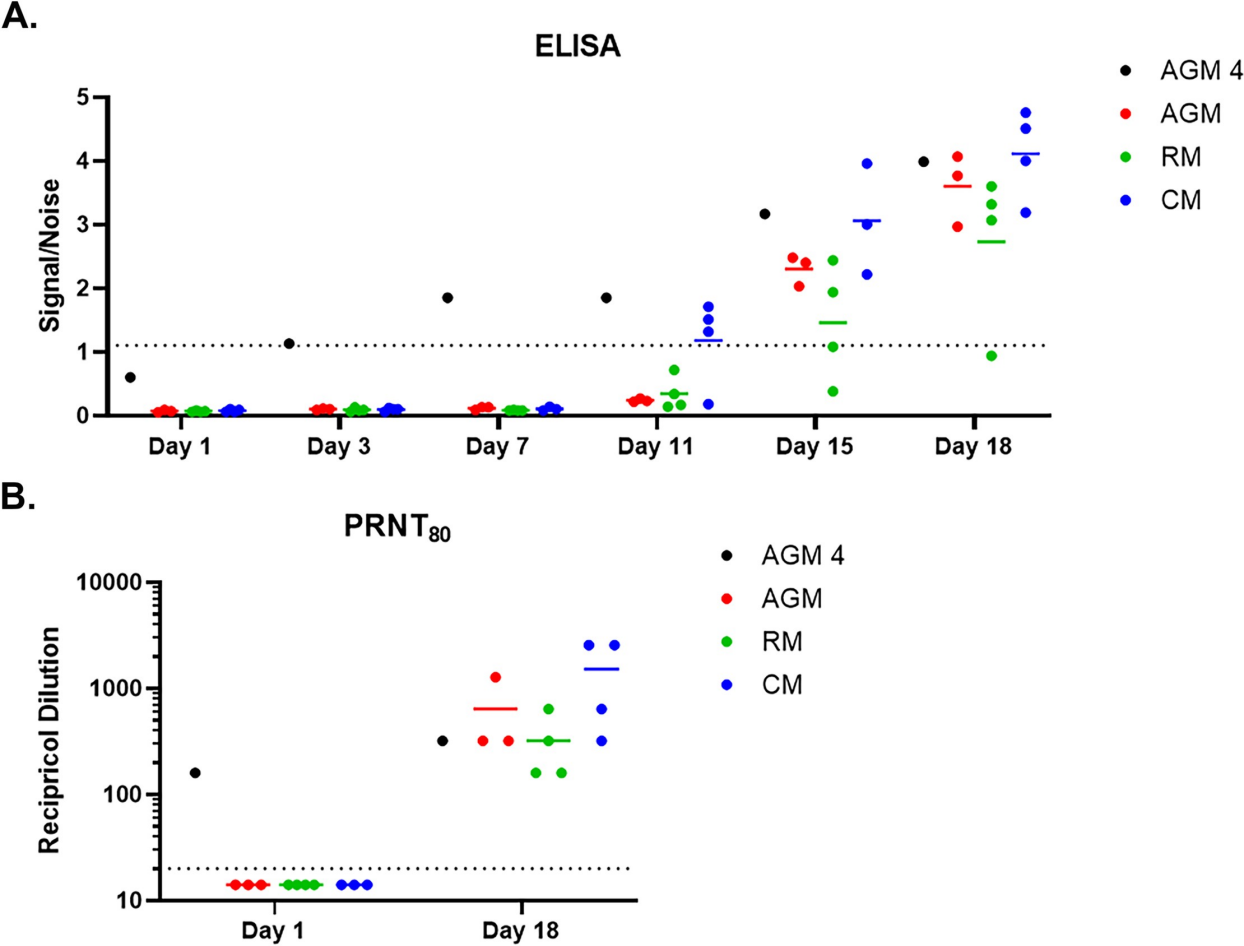
Characterization of immune response using ELISA and PRNT. A. Serum samples were screened by commercial Euroimmun SARS-CoV-2 S1 IgG ELISA kit. This direct ELISA kit measures IgG response against the S1 subunit of the spike glycoprotein. The dotted line represents the assay cutoff, above which a sample is considered above background noise (i.e. positive). B. The PRNT_80_ titer is shown, and the dotted line represents the assay cutoff, above which a sample is considered above background noise (i.e. positive).

**Fig 7:**
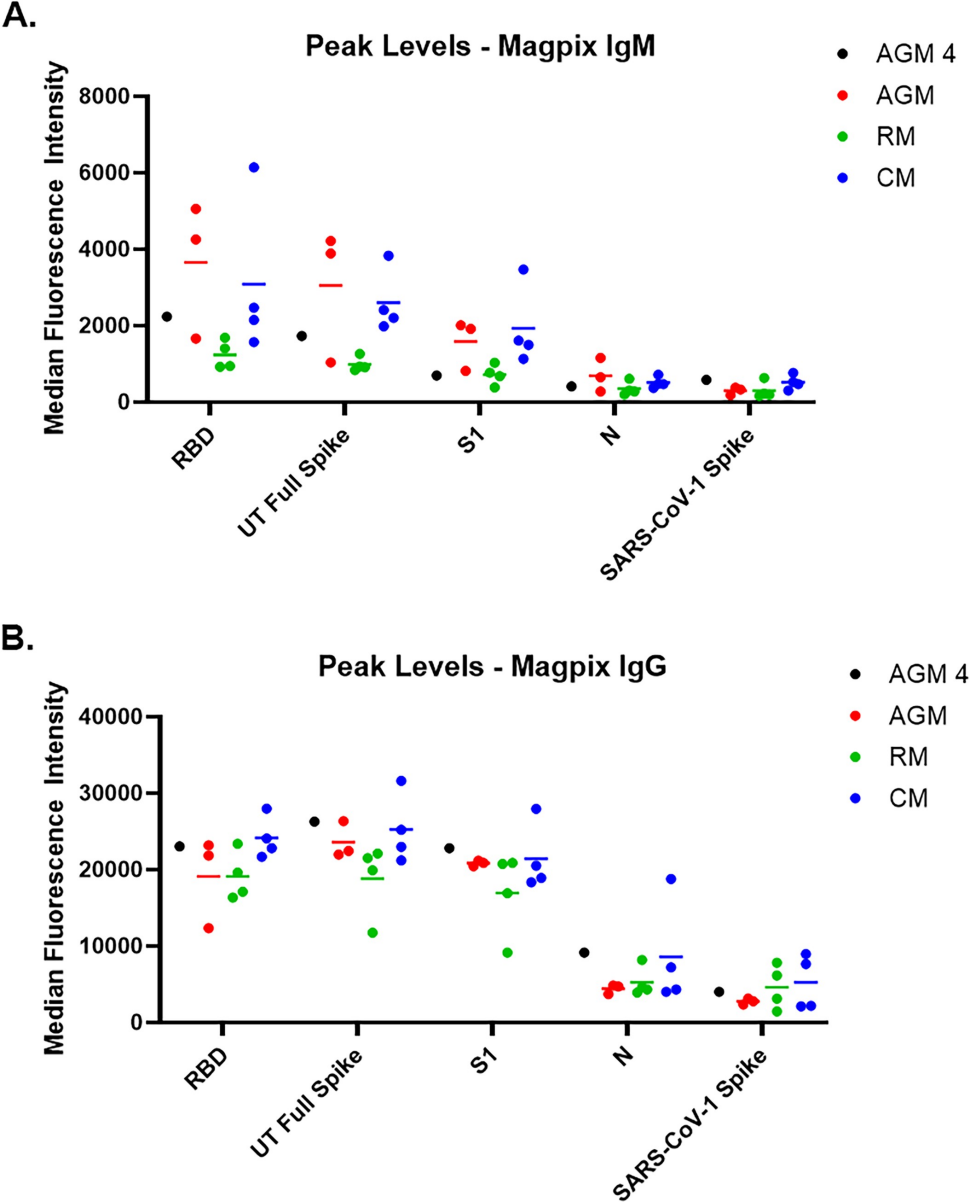
Characterization of immune response using a Magpix multiplex immunoassay. Peak response for IgM (A) and IgG (B) to various antigens using the Magpix multiplex bead-based immunoassay is shown. Longitudinal Magpix data can be found in S3 Fig.

As previously mentioned, all study-assigned NHP were serologically negative for SARS-CoV-2 prior to virus challenge, and this was demonstrated in both the Euroimmun ELISA assay as well as the PRNT. Furthermore, these animals developed a characteristic immune response following infection. An IgG response in the Euroimmun ELISA was detected by Study Day 11 for CMs, and Study Day 15 for AGMs and RMs, with levels continuing to increase to end-of-study (Fig 6). Additional characterization of the immune response using the Magpix multiplex immunoassay revealed both an IgM and IgG response to SARS-CoV-2 (Fig 7 and S3 Fig). IgM signals to SARS-CoV-2 glycoprotein antigens were detected as early as Study Day 9 for AGMs and RMs, and Study Day 7 for the CMs; IgM signals to SARS-CoV-1 spike and SARS-CoV-2 nucleoprotein were not detected, demonstrating the specificity of the assay. IgG signals were also detected by Study Day 9 for AGMs and RMs, and Study Day 7 for CMs. Unlike IgM assay, the IgG Magpix assay seemed to be slightly less specific for SARS-CoV-2 glycoprotein antigens, detecting to varying degrees SARS-CoV-1 spike and SARS-CoV-2 nucleoprotein antigens. PRNT assays were also performed on post-challenge samples (Fig 6). A PRNT_80_ of less than 1:20 at time of challenge was used to confirm study-enrolled animals were serologically negative for SARS-CoV-2, whereas PRNT performed on terminal (end-of-study) samples revealed a strong neutralizing antibody response, with PRNT_80_ titers between 1:160 and 1:2560.

A fourth AGM (AGM 4) was screened for inclusion in this study. However, qRT-PCR analysis on a pre-study nasopharyngeal swab revealed that this animal was positive for SARS-CoV-2 (of note, none of the nasopharyngeal swabs collected from other study enrolled animals were positive pre-study). 17 segregating sites (SNPs) were detected between the Washington Isolate (MT020880.1) and the AGM infection sampled (S4 Fig). This sequence (Genbank Accession # SUB8892677 LN_Apr6_RNA_access MW477798) and the sequence of the viral isolate utilized by USAMRIID are from different phylogenetic clades (S5 Fig). Other phylogenetically similar sequences to the 6 APR 20 sample are observed in the US (Arizona, Georgia, Virginia) and in Europe (Greece) (S6 Fig) indicate a probable circulation in the Maryland area during the time in question. This transmission event most likely occurred from an importation from Europe into a neighboring US port of entry. The most probable introduction of infection appears to be community based. The NHP was, therefore, excluded from the main study. However, due to the importance of understanding the effects of prior exposure on disease pathogenesis following re-infection with SARS-CoV-2, this animal was still experimentally exposed to SARS-CoV-2 alongside the other AGM, with special attention paid to differences in viral RNA load in NP swabs and immunological responses compared to other AGM. It should be noted that, aside from the PCR finding, this animal was clinically healthy on the day of exposure.

**Fig 8:**
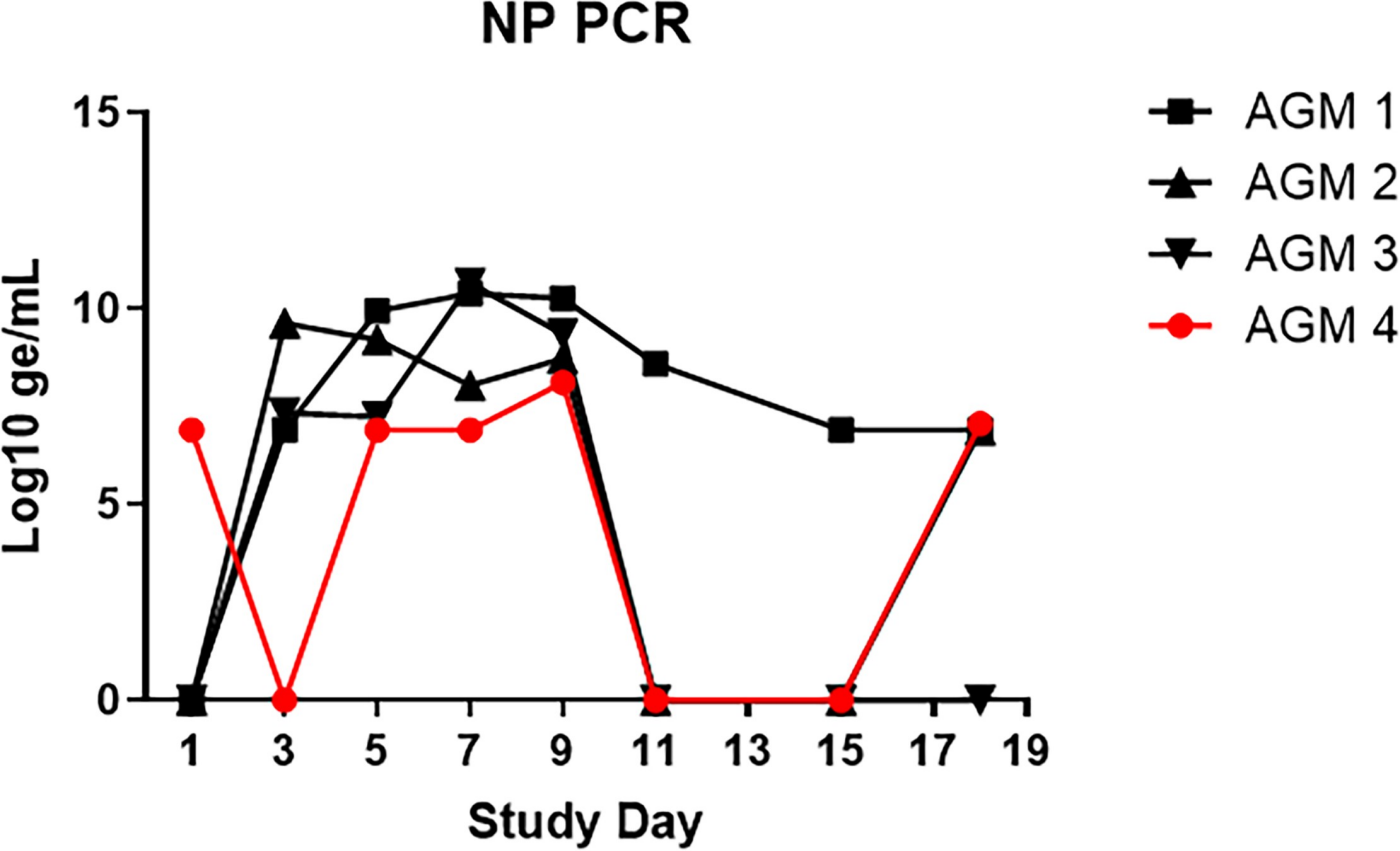
Viral RNA in nasopharyngeal swabs for AGM 4. SARS-CoV-2-specific qRT-PCR was performed on RNA extracted from nasopharyngeal swab clarified homogenates. Data are shown as Log_10_ ge/mL. For comparison, data for AGM 1–3 are also shown.

PCR data collected on NP swabs from challenge day confirmed that AGM 4 did have a current or recent active infection with SARS-CoV-2, but by Study Day 3, (when viral RNA was first detected in NP swabs for other AGM) this animal had no detectable viral RNA in the NP swab (Fig 8). By Study Day 5, however, AGM 4 again had detectable viral RNA in NP swabs, with levels peaking at 8.8 Log_10_ ge/mL by Study Day 9. Although levels for this animal remained 1–2 logs lower than other AGM, the presence of high levels of viral RNA in NP swabs is evidence of likely re-infection with SARS-CoV-2. This is corroborated by the fact that this NHP also developed clinical signs of disease, similar to AGMs 1–3, between 3–18 days following experimental challenge; these signs included increased respiratory sounds on auscultation, lymphadenopathy, evidence of increased respiratory effort (i.e. abdominal component to breathing), and radiographic findings of lung opacity and infiltrates.

The reason for the reduced viral RNA load in NP swabs for AGM 4, as well as the delayed detection after challenge day compared to other AGMs, was likely an ongoing immunological response mounted against the community-acquired SARS-CoV-2 infection. AGM 4 showed an early immunological response, with IgM detected on the day of challenge, and IgG detected by Study Day 3 (Figs 6 and 7 and S3 Fig). Antibody levels appeared to plateau around Study Day 7; however, an anamnestic response for IgG was observed on Study Day 15, with levels increasing through end-of-study. Peak levels of IgG at end-of-study were similar for all AGM, including AGM 4.

ELISA and Magpix data for AGM 4 were further confirmed in the PRNT assays (Fig 6). This animal had neutralizing antibodies against SARS-CoV-2 at the time of challenge that were largely unchanged at end-of-study.

## Discussion

Due to the ongoing pandemic of COVID-19, it is critical that relevant animal models that recapitulate human disease characteristics are developed to facilitate immediate vaccine and treatment testing. Nonhuman primate models will be key to this testing, as data generated will be combined with human clinical data to support rapid deployment of safe and efficacious countermeasures for human use. Herein, we describe the evaluation of the first airborne SARS-CoV-2 model in macaques, and compare the results to the previously-described airborne model in AGMs [17, 18]. The development of macaque models is critical to advance countermeasure testing efforts. Although prior studies have highlighted the potential utility of AGMs for SARS-CoV-2 testing, certain characteristics might make these nonhuman primates less than ideal candidates. AGMs are generally wild-caught and imported for use on study; the medical history for these animals is typically only available from the time of import. In addition, age can only be approximated, as actual birth dates for AGMs are generally unavailable. Finally, wild-caught primates represent a diverse genetic population, resulting in inconsistent cohorts, which may include animals predisposed to developing more severe disease following viral infection. For this reason, AGMs are not typically chosen as gold-standard models of viral disease. In contrast, CMs and RMs have represented critical models for countermeasure development for various pathogens over many years. This is because this population of animals is more uniform as they can be acquired from breeding colonies. Health and age information is generally available from the time of birth, decreasing the likelihood of outliers based on genetic or medical background. It is for this reason that we sought to investigate airborne exposure with SARS-CoV-2 in macaque models.

The airborne model mimics a predicted natural transmission route [13, 19–22], compared to other nonhuman primate models of COVID-19 which rely on direct contact inoculation of mucosal and respiratory surfaces. Exposure of AGM, RM, and CM to SARS-CoV-2 resulted in disease similar to mild cases of human COVID-19. Increased lung sounds on auscultation in association with radiographic findings consistently confirmed the presence of mild to moderate respiratory disease for all animals on study, and febrile illness was achieved for all CM.

CM developed a consistent disease, with fever being a hallmark of disease exclusive to this group. Lung infiltrates, visualized radiographically, were also common for animals in this group. The clinical disease severity score for CM was 3.00 points higher than AGM and 4.25 points higher than RM. Elevation of more than one liver-related enzyme and serum glucose were also consistently measured for CM. Overall, there were sufficient objective criteria for this group that could be easily employed to evaluate efficacy in future countermeasure efforts.

CMs have been investigated as a potential model for SARS-CoV-2, with variable disease findings noted following either IT/IN or IT/IN/ocular inoculation [5, 23]. Importantly, fever, which represents a prominent human disease sign and which was consistently noted for CM on the present study, was not noted for any CMs on these prior studies. In addition, many of the other clinical signs of disease observed in the present study for CM were not reported for the prior studies, aside from infiltrates noted radiographically in only one of those studies. Therefore, using an airborne transmission route, we were able to generate a model of COVID-19 in CM that was more representative of human disease compared to prior work utilizing this species.

The AGM aerosol model of SARS-CoV-2 infection has been recently defined [17, 18, 24]. In general, findings in the present study were similar to those previously described. There were two key hematologic findings that make this an attractive model, thrombocytopenia and monocytosis. Thrombocytopenia has been noted during human disease [14], following IT/IN inoculation [24], and following aerosol exposure of AGM [18], and this finding was consistently noted for AGM on this study. Similarly, pronounced elevation of monocytes which far exceeded levels for the other species was consistently noted for AGM. This finding has not been previously reported; the physiological relevance is unknown at this time and warrants further investigation.

Blair *et al* have been the only group thus far to describe acute respiratory distress syndrome in nonhuman primates following SARS-CoV-2 inoculation, and this was observed in only two AGM, one exposed to virus by the aerosol route and the other exposed via a combination route (conjunctival/IT/oral/IN) [17]. It should be noted that the animals used on that particular study were “aged”. In addition, only two animals were evaluated for each exposure route, bringing into question the relevance of the finding (can severe COVID-19 actually be achieved using aged nonhuman primates, or were the two animals that developed this finding simply outliers with underlying and undetected age-related conditions that predisposed them to more severe disease).

RM have been the most extensively used SARS-CoV-2 model species, with the IT/IN or IT/IN/ocular/oral RM models being the gold standards for use in countermeasure efficacy studies [5–7]. Clinical disease following airborne virus exposure was similar to that reported following IT/IN or IT/IN/ocular/oral inoculation. RM developed a mild respiratory disease characterized by increased respiratory noise on auscultation and changes in lung opacity radiographically. Similar to AGMs, thrombocytopenia was noted for animals in this group. Erythema around the eyes was a consistent and nearly exclusive finding for animals in this group; however, this is a relatively subjective measurement and may have been the result of ocular exposure during challenge (the eyes were not covered during airborne challenge). Rectal bleeding was another reliable finding for RM; however, this finding was most likely related to repeated swabbing, resulting in irritation of the rectal tissue, and not a SARS-CoV-2-specific finding.

One of the most consistent objective findings in the literature for AGM, CM and RM has been detection of high levels of viral RNA in NP and OP swab samples. SARS-CoV-2 RNA peak levels and kinetics in these studies were similar to those measured on the present study, regardless of route of inoculation, virus variant, inoculation dose, or species of primate used [5–7, 17, 18, 23, 24]. This information is critical in that it demonstrates that measurement of SARS-CoV-2 RNA by RT-PCR represents a reliable and reproducible endpoint that can be employed across testing facilities for countermeasure efficacy determinations.

Nearly all animals on the present study had some degree of inflammation and chronic lesions in one or more lung lobes at time of necropsy, and these findings were consistent with those previously reported for direct contact inoculation models [5–7, 23, 24]. Alveolar fibrosis and type II pneumocyte hyperplasia indicative of previous cellular damage were more frequently noted for AGM and CM. By Study Day 18, most clinical disease signs (including radiographic findings) had either completely resolved or were resolving, and virus in lung tissue was below the limit of detection by ISH. Prior studies have demonstrated the ability to consistently detect SARS-CoV-2 virus by immunohistochemistry of ISH in formalin-fixed tissues collected prior to Study Day 10 [25–27]. However, similar to the data described herein, assessment of tissues by these methods after Study Day 10 typically leads to negative results. Therefore, it may be important to investigate whether necropsy performed earlier in disease course could be beneficial to assessing the active disease state of primates infected by SARS-CoV-2. For countermeasure testing, where delayed disease is a possibility, the decision to shorten the length of study would obviously also need to be balanced against the risk of missing delayed-onset disease.

In cases where survival cannot be used as a primary endpoint for countermeasure efficacy determinations, other statistically relevant findings are necessary, with objective criteria being preferred over subjective criteria. Herein, we describe two models (CM and AGM) for which obvious and consistent objective criteria exist that could be used for future countermeasure studies as statistical endpoints. For CM, these endpoints could be fever, the presence of lung infiltrates radiographically, and shed virus in NP and/or OP swabs. For AGM, these endpoints could be platelet levels, monocyte levels, and shed virus in NP and/or OP swabs. Although RM had fewer objective findings compared to CM and AGM, consistent subjective findings associated with shed virus in NP and/or OP swabs still make this a very valuable and utilizable COVID-19 model. Regardless of species, the present study demonstrated that macaques could be successfully infected by airborne SARS-CoV-2. Considering relevance to human disease, this study demonstrated that airborne nonhuman primate models should be strongly considered for any future countermeasure evaluations. The species to be used for such studies should be carefully considered based on desired endpoints; however, any of the species described herein could be used successfully for pathogenesis studies as well as for the testing of vaccines, antivirals, and therapeutics.

The study also demonstrated the importance of thorough pre-screening of NHP to be used on SARS-CoV-2 studies. Due to the high transmissibility rate of COVID-19, workflow and PPE considerations must be taken to mitigate pre-exposure risk of NHP colonies to fomites or other potentially infectious exposure routes. Even under the most stringent PPE stance, this study demonstrated that human-to-primate transmission can occur, and pre-exposure can affect disease characteristics and immunological responses. In a study previously conducted in RM, Deng *et al* showed that animals exposed to SARS-CoV-2 are completely protected against reinfection with the same pathogen [28]. In their study, viral RNA was not detected in NP swabs from re-infected RM, and these animals were also free of any clinical signs of disease. In the present study, AGM 4, which had a prior exposure to a circulating strain of SARS-CoV-2, did develop signs of disease and other evidence of re-infection (including quantifiable viral RNA levels in swab samples) post experimental challenge. It should be noted that although our findings for AGM 4 somewhat contradict previous findings in re-infected RM, the information from both studies is based on very small numbers of animals and in no way should be used as evidence or proof of the reinfection capability of SARS-CoV-2. However, what the present study did conclusively demonstrate was the importance of comprehensive pre-screening of animal cohorts to be used for countermeasure evaluations, and of implementing measures to minimize the unintentional community to cohort virus transfer, as the data clearly show that SARS-CoV-2 pre-exposure would likely have a dramatic impact on any evaluation of vaccine or therapeutic efficacy and protective response.

## Materials and methods

### Animals

Animal research was conducted at the United States Army Medical Research Institute of Infectious Diseases (USAMRIID). Three adult *Chlorocebus aethiops* (African green monkeys) of Caribbean origin, four adult *Macaca mulatta* (rhesus macaques) of Chinese origin, and four adult *Macaca fascicularis* (cynomolgus macaques) of Chinese origin were included on this study. A fourth AGM was screened for inclusion in this study. However, qRT-PCR analysis on a pre-study nasopharyngeal swab revealed that this animal was positive for SARS-CoV-2. It was concluded that this animal had a community-acquired infection and the NHP was, therefore, excluded from the main study. However, due to the importance of understanding the effects of prior exposure on disease pathogenesis following re-infection with SARS-CoV-2, this animal was still experimentally exposed to SARS-CoV-2 alongside the other AGM. Cynomolgus macaques and rhesus macaques were between 5–10 years old at time of challenge. African green monkeys are wild caught so exact ages are not known, but based on the time in the colony it is assumed that these animals were at least 3 years old at time of challenge. Genders were mixed male and female, and all animals were SARS-CoV-2 serologically naïve. All animals had passed a semi-annual physical examination and were certified as healthy by a veterinarian. Animals were acclimated in ABSL-3 animal rooms for 3 days prior to virus exposure and housed individually in 4.3 square foot cages. During the in-life portion of the study, animals were provided 2050 Monkey Chow (Harlan Teklad, Frederick, MD), fruits, and water ad libitum via an automatic watering system, and animals were given enrichment regularly as recommended by the Guide for the Care and Use of Laboratory Animals.

#### Ethics statement

These experiments and procedures were reviewed and approved by the United States Army Medical Research Institute for Infectious Diseases Institutional Animal Care and Use Committee (IACUC). All research was conducted in compliance with the USDA Animal Welfare Act (PHS Policy) and other federal statutes and regulations relating to animals and experiments involving animals, and adheres to the principles stated in the Guide for the Care and Use of Laboratory Animals, National Research Council, 2011. The facility is fully accredited by the Association for Assessment and Accreditation of Laboratory Animal Care, International. The animals were provided food and water ad libitum and checked at least daily according to the protocol. All efforts were made to minimize painful procedures; the attending veterinarian was consulted regarding painful procedures, and animals were anesthetized prior to phlebotomy and virus infection. Animals were humanely euthanized at the end of study by intracardiac administration of a pentobarbital-based euthanasia solution under deep anesthesia in accordance with current American Veterinary Medical Association Guidelines on Euthanasia and institute standard operating procedures.

### Virus and virus exposure

A seed stock of SARS-CoV-2, isolate 2019-nCoV/USA-WA1/2020, designated as Lot R4717, was grown on ATCC Vero 76 cells using Stock Lot R4716 [Centers for Disease Control and Prevention (CDC) obtained]. The seed stock contains an average of 1.56x10^6^ pfu/mL of infectious virus as determined using a standardized agarose and neutral red-based assay. R4717 was fully sequenced, evaluated for sterility, tested for mycoplasma and endotoxin levels, and tested in a number of real-time reverse transcriptase polymerase chain reaction (RT-PCR) assays to include two specific for SARS-CoV-2 virus. It was determined to have no detectable mycoplasma, endotoxin or adventitious agents based on the assays and techniques used. No known contaminants were detected when sequencing the stock. Identity was confirmed by real-time RT-PCR. This stock does not contain any known contaminants and was deemed appropriate for use in testing for coronavirus therapeutics screening. On the day of exposure, Study Day 1, animals were exposed to undiluted R4717 in the USAMRIID head-only exposure system. The aerosol spray is generated using a Collison Nebulizer to produce a highly respirable aerosol (flow rate 7.5±0.1 L/minute). The system generates a target aerosol of 1 to 3 um mass median aerodynamic diameter determined by aerodynamic particle sizer. Samples of the aerosol collected from the exposure chamber using an all-glass impinger during each exposure were assessed using a plaque assay. The exposure dose for each animal was calculated from the minute volume determined with a plexiglass whole body plethysmograph box using Buxco FinePointe software. The total volume of aerosol inhaled was determined by the exposure time required to deliver the estimated inhaled dose.

### Animal observations

Animals were evaluated cage side for signs of illness. Other observations such as biscuit/fruit consumption, condition of stool, and urine output were also documented, if possible. Observations under anesthesia (physical examinations) occurred after cage side observations on Study Days 1, 3, 5, 7, 9, 11, 15, and the day of disposition (i.e. Study Day 18). Body weights, pulse oximetry, auscultation of heart and lung sounds, radiography, blood collection, and collection of swab samples occurred during physical examinations.

### Telemetry

Telemetry implants (M00; Data Sciences International, St. Paul, MN) were used to continuously monitor body temperature and activity in subject animals. Subjects were housed in individual cages in close proximity to radio frequency digital transceivers (TRX; Data Sciences International, St. Paul, MN) equipped with directional antennas pointed at the animal cages. These transceivers were connected via cat5e cables to a set of Communication Link Controllers (CLC, Data Sciences International, St. Paul, MN) to allow the digital multiplexing and the simultaneous collection of signals from all subjects. The signals were then routed over cat5e cable to data acquisition computers, which captured, reduced and stored the digital data in data files (i.e., NSS files) using the Notocord-hem Evolution software platform (Version 4.3.0.77, Notocord Inc., Newark, New Jersey). Reduced data in the NSS files was extracted into Microsoft Excel workbooks using Notocord-derived formula add-ins, and the 30-minute (min) averages were calculated for each parameter for each subject. Telemetry data collected prior to challenge was used as baseline, and provided the average and standard deviation (SD) for each 30 min daily time of a 24-hour day.

### Necropsy, histology, *in situ* hybridization, and immunofluorescence

Necropsies were conducted by a veterinary pathologist on all animals in this study. The tissue samples were trimmed, routinely processed, and embedded in paraffin. Sections of the paraffin-embedded tissues 5 um thick were cut for histology. For histology, slides were deparaffined, stained with hematoxylin and eosin (H&E), coverslipped, and labeled. *In situ* hybridization was performed as previously described [29].

For immunofluorescence, the following procedures were performed. After deparaffinization and treatment with 0.1% Sudan Black B to reduce autofluorescence, tissues were heated in citrate buffer, pH 6.0 (Sigma-Aldrich, St. Louis, MO), for 15 min to reverse formaldehyde cross-links. After rinses with phosphate-buffered saline (PBS), pH 7.4 (Thermo Fisher Scientific, Waltham, MA), sections were blocked overnight with PBST (PBS+ 0.1% Tween-100) containing 5% normal goat serum (MilliporeSigma, Temecula, CA) at 4°C. Sections were then incubated with the following primary antibodies for 2 h at room temperature: rabbit polyclonal antibody against Ki67 at a dilution of 1:400 (ab15580, Abcam, Waltham, MA); rabbit polyclonal anti-CD3 antibody at a dilution of 1:200 (A045229-2, Dako Agilent Pathology Solutions, Carpinteria, CA); mouse anti-human CD68 antibody at a dilution of 1:200 (Clone KP1, Dako Agilent Pathology Solutions, Carpinteria, CA); mouse monoclonal antibody against CD45 at a dilution of 1:200 (Clone 2B11 + PD7/26, Dako Agilent Pathology Solutions, Carpinteria, CA). After rinsing in PBST, sections were incubated with secondary goat IgG Alexa Fluor 488-conjugated anti-rabbit and with goat IgG Alexa Fluor 561-conjugated anti-mouse antibody (Thermo Fisher Scientific, Waltham, MA) for 1 h at room temperature. Sections were cover-slipped using VECTASHIELD antifade mounting medium with DAPI (Vector Laboratories, Burlingame, CA). Images were captured on an LSM 880 Confocal Microscope (Zeiss, Oberkochen, Germany) and processed using open-source ImageJ software (National Institutes of Health, Bethesda, MD).

### Clinical pathology

For serum chemistries, whole blood was collected into Serum Clot Activator Greiner Vacuette tubes (Greiner Bio-One, Monroe, NC). Tubes were allowed to clot for at least 10 min and the serum separated in a centrifuge set at 1800 × g for 10 min at ambient temperature. The required volume of serum was removed for chemistry analysis using a General Chemistry 13 panel (Abaxis, Union City, CA) on a Piccolo Point-Of-Care Analyzer (Abaxis, Union City, CA). Serum was removed from the clot within 1 hour of centrifugation and was analyzed within 12 hours of collection.

For hematology, whole blood was collected into Greiner Vacuette blood tubes containing K3 EDTA as an anti-coagulant. Hematology was performed on VETSCAN® HM5 hematology analyzer (Abaxis, Union City, CA) within 4 hours of collection. In addition, 100 uL of whole blood was added to 300 uL of TRIzol® LS (Thermo Fisher Scientific, Waltham, MA) for RNA isolation for qRT-PCR.

### Swab specimen processing

Swab samples were suspended in 1 mL of viral transport media (Hanks Balanced Salt Solution containing 2% heat-inactivated fetal bovine serum, 100 ug/mL gentamicin, and 0.5 ug/mL amphotericin B) by vortex for 15–20 seconds followed by incubation at 2–8°C for 20–25 min. Following another 15–20 second vortex, clarification was performed by centrifugation at 14,000 rpm for 30 seconds, and 100 uL of clarified supernatant was added to 300 uL of TRIzol® LS for RNA isolation for qRT-PCR. In addition, 200 uL of clarified supernatant was analyzed for infectious virus by plaque assay.

### qRT-PCR

TRIzol LS whole blood and swab specimens were extracted and eluted with AVE buffer using a QIAamp® Viral RNA Mini Kit (Qiagen, Germantown, MD). The RT-PCR reaction used Invitrogen™ SuperScript® One-Step RT-PCR System with additional magnesium sulfate (MgSO_4_) added to a final concentration of 3.0 mM. Specimens were run in triplicate using a 5-uL volume. The average of the triplicates were multiplied by 200 to obtain genomic equivalents per mL, then multiplied by a dilution factor of 4 (1 part clarified supernatant to 3 parts TRIzol LS) for the final reported value. The genomic equivalents were determined using a standard curve of synthetic RNA of known concentration. Sequences of primers and probes used are as follows:

Forward primer sequence (5’-3’): TTACAAACATTGGCCGCAAA
Reverse primer sequence (5’-3’): GCGCGACATTCCGAAGAA
Probe sequence (5’-3’): ACAATTTGCCCCCAGCGCTTCAG

### Plaque assay

Challenge dose and infectious virus in swab samples were determined by agarose plaque assay, run on fresh (i.e. not frozen then thawed) samples the day of collection. Required dilutions of each specimen were prepared in virus diluent [1X minimum essential media (Corning Life Sciences, Pittston, PA) containing 10% fetal bovine serum (Cytiva Life Sciences, Marlborough, MA), 1% GlutaMAX (Gibco, Waltham, MA), and 1% NEAA (Sigma, St. Louis, MO)] in duplicate (swab specimens) or triplicate (all-glass impinger specimens from Study Day 1), were added to plates containing Vero 76 cells (ATCC, Manassas, VA) at greater than or equal to 85% confluency. Two days later, the cells were stained with neutral red (Thermo Fisher Scientific, Waltham, MA), and plaque counts were obtained the day after staining.

### Euroimmun SARS-CoV-2 S1 ELISA

NHP serum samples were screened with the Euroimmun SARS-CoV-2 S1 ELISA kit (Euroimmun, EI 2606–9601 G, Mountain Lakes, NJ) per manufacturer’s instructions. Briefly, the kit materials were brought to ambient temperature for 30 min. Serum samples were diluted 1:101 using the supplied sample buffer. A volume of 100 uL of the diluted samples, supplied controls, and supplied calibrator were added to the pre-coated wells and incubated at 37°C for 1 hour. The plate was washed 3 times with 300 uL of supplied wash buffer using a microplate washer (Biotek 405TS, Winooski, VT). A volume of 100 uL of enzyme conjugate was added to the wells, and the plate was incubated at 37°C for 30 min. The plate was washed 3 times as outlined above prior to adding 100 uL of substrate for 30 min at ambient temperature. Finally, 100 uL of stop solution was added prior to reading absorbance at 450 nm, with a reference wavelength at 635 nm (Tecan M200, Männedorf, Switzerland). Data was processed according to kit instructions to determine negative, positive, or borderline results.

### Plaque reduction neutralization test

An equal volume of complete media [Eagle’s Minimum Essential Media (EMEM, Corning, Tewksbury, MA) containing 10% heat inactivated fetal bovine serum (HI-FBS, Cytiva, Marlborough, MA), 10mM HEPES (Sigma, St. Louis, MO), 1% Penicillin/Streptomycin (ThermoFisher, Carlsbad, CA, 0.1% gentamycin (Sigma), and 0.2% fungizone (Sigma)] containing SARS-CoV-2 was combined with 2-fold serial dilutions of NHP serum in complete media (total volume 222 microliters), and the mixture was incubated at 37°C in a 5% CO_2_ incubator for 1 hour. The combined virus/antibody mixture was then added to Vero 76 (ATCC, Manassas, VA) cell monolayers in 6-well plates (180 uL per well) and allowed to adsorb for 1 hour at 37°C. Agarose overlay [Basal Medium Eagle with Earle's salts (EBME,) with HEPES containing 0.6% SeaKem ME agarose (Lonza, Rockland, ME), 100X nonessential amino acids (ThermoFisher), 1% Penicillin/Streptomycin (ThermoFisher), 0.1% Gentamycin (Sigma), and 0.2% fungizone (Sigma)] containing 10% HI-FBS was then added (3 mL per well) and allowed to solidify at ambient temperature. The plates were placed in a 37°C incubator for 2 days, and then agarose overlay containing 5% neutral red (ThermoFisher) and 5% HI-FBS was added (2 mL per well) and the plates were returned to 37°C. After 1 day, the number of plaques were counted, and the PRNT_80_ titers were calculated as the reciprocal of the highest dilution that resulted in a 80% reduction in the number of plaques relative to virus alone.

### Magpix multiplex immunoassay

Recombinant SARS-CoV-2 full trimeric spike (gift from Dr. Jason McLellan’s group; UT-Austin [30]), S1 (Sino Biological, 40591-V08H, Chesterbrook, PA), RBD (Sino Biological, 40592-V08H), NP (Native Antigen Company, REC31812-100, Kidlington, UK), and SARS-CoV-1 full spike (Protein Sciences, Meriden, CT) proteins were conjugated to magnetic microspheres using the Luminex xMAP® antibody coupling kit (Luminex Inc., Austin, TX) according to the manufacturer’s instructions. Briefly, 500 uL of Magplex microspheres (12.5x10^6^ microspheres/mL) were washed three times using a magnetic microcentrifuge tube holder, and resuspended in 480 uL of activation buffer. Then, 10 uL of both sulfo-N-hydroxysulfosuccinimide (sulfo-NHS) and 1-Ethyl-3-[3-dimethylaminopropyl]carbodiimide hydrochloride (EDC) solutions were added to the resuspended microspheres. The tube was covered with aluminum foil and placed on a benchtop rotating mixer for 20 min. After surface activation with EDC, the microspheres were washed three times with activation buffer prior to adding the recombinant protein antigen at a final concentration of 4 ug antigen:1x10^6^ microspheres. This concentration of recombinant protein coupled to the surface of microspheres has been shown to be optimal for IgG and IgM detection [31]. The tube was again covered with aluminum foil and placed on a benchtop rotating mixer for 2 hours. After this coupling step, the microspheres were washed three times with wash buffer and resuspended in 500 uL of wash buffer for future use. SARS-CoV-2 full spike, S1, RBD, NP, and SARS-CoV-1 full spike were coupled to Magplex microsphere regions #45, #55, #65, #25, and #77, respectively, in order to facilitate multiplexing experiments. Beads were stored at 4°C until further use.

NHP serum samples were diluted 1:100 in 1X PBS containing 0.02% Tween-20 (Sigma, St. Louis, MO) (PBST) and 5% skim milk (PBST-SK). Serial 1:3 dilutions were made starting at 1:900. Each individual antigen-coupled bead was mixed at a 1:1 ratio prior to diluting in PBST to 5x10^4^ microspheres/mL, and the mixture was added to the wells of a Costar polystyrene 96-well plate at 50 uL per well (2500 microspheres of each antigen bead set/well). The plate was placed on a magnetic plate separator (Luminex Corp., Austin, TX), covered with foil, and microspheres were allowed to collect for 60 sec. While still attached to the magnet, the buffer was removed from the plate by inverting. Then, 50 uL of diluted serum samples were added to appropriate wells. The plate was covered with a black, vinyl plate cover and incubated with shaking for 1 hour at ambient temperature. The plate was washed three times with 100 uL of PBST for each wash, using the plate magnet to retain the Magplex microspheres in the wells. Liquid was discarded by inverting as above. Next, 50 uL of a 1:100 dilution of mouse anti-human IgM phycoerythrin conjugate (Invitrogen, MA1-10381, Carlsbad, CA) or goat anti-human IgG phycoerythrin conjugate (Sigma, P9170) in PBST-SK was added to the wells. The plate was covered again with a black, vinyl plate sealer and incubated with shaking for 1 hour at ambient temperature. After incubation, the plate was washed three times as detailed above, and the Magplex microspheres were resuspended in 100 uL of PBST for analysis on the Magpix instrument (Luminex Corp). Raw data was reported as median fluorescence intensity for each bead set in the multiplex.

### Genome sequencing analysis

Raw sequences from the MiSeq (Illumina, San Diego, CA) were cleaned and aligned using an in-house perl wrapper, VSALIGN, developed by the United States Army Medical Research Institute of Infectious Diseases Center for Genomic Sciences. VSALIGN performs read cleaning and quality control using PRINSEQ-lite and in-house scripts. This cleaning and pre-processing step included adapter removal, duplicate/chimeric read removal, and quality filtering, keeping reads with an average quality score > 25 (phred). Pre-processed and filtered reads were aligned to the strain MT020880.1, which the USAMRIID stock is derived from, using Lasergene nGen (DNAStar, Madison, WI). Changes relative to the MT020880.1 sequence required greater than 50% of the reads supporting the change, with at least 20x coverage.

SARS-CoV-2 sequences were aligned with MAFFT v7.220 using default settings followed by manual curation using Geneious version R9 (Biomatters, Ltd., Auckland, New Zealand). The statistical selection of the best-fit nucleotide substitution model was performed with jModelTest2 [32, 33]. We reconstructed a Maximum-likelihood tree based on the GTR+Γ4 model using the software FastTree v2.1 (http://www.microbesonline.org/fasttree/) under an exhaustive search, and computing 5000 local support values (Shimodaira-Hasegawa test).

## Supporting information

S1 Fig Clinical disease severity. Disease severity based on clinical signs was graded using a scoring system that can be found in S1 Table.
(TIF)

S2 Fig Viral RNA and live virus in rectal swabs. SARS-CoV-2-specific qRT-PCR was performed on RNA extracted from nasopharyngeal and oropharyngeal swab clarified homogenates. Plaque assay was performed on nasopharyngeal and oropharyngeal clarified homogenates. Data are shown as Log_10_ ge/mL (qRT-PCR) or Log_10_ pfu/mL (plaque assay).
(TIF)

S3 Fig Longitudinal IgM and IgG response. The longitudinal IgM and IgG response against SARS-CoV-2 UT full spike as measured by Magpix multiplex immunoassay is shown. Average = the average of the median fluorescence intensity for the indicated antibody response for CMs, RMs, and AGMs 1–3.
(TIF)

S4 Fig Sequencing analysis diagram #1. A visual depiction of sequence similarity with highlighted changes between the viral stocks available at USAMRIID and the April 6th sample during the time of the study.
(PDF)

S5 Fig Sequencing analysis diagram #2. A radial Maximum-likelihood tree estimated using 1,003 SARS-CoV-2 coding-complete genomes. The WA1 strain (Genbank accession number: MT020880) and consensus genome sequence from an infected non-human primate nasal swab collected on April 6th are highlighted using colored circles. Tree branches are scaled by substitutions per site. Tree node support values were generated using 5000 Shimodaira-Hasegawa tests and are shown in decimal form.
(PDF)

S6 Fig Sequencing analysis diagram #3. A close view of the near neighbors to the sequence generated April 6th sample.
(TIF)

S1 Table Nonhuman primate COVID-19 clinical disease severity. (DOCX)

S2 Table Summary clinical pathology findings. (DOCX)

## References

[1] Chen N, Zhou M, Dong X, Qu J, Gong F, Han Y, et al Epidemiological and clinical characteristics of 99 cases of 2019 novel coronavirus pneumonia in Wuhan, China: a descriptive study. Lancet. 2020;395(10223):507–13. Epub 2020/02/03. 10.1016/S0140-6736(20)30211-7 . 32007143 PMC7135076

[2] Huang C, Wang Y, Li X, Ren L, Zhao J, Hu Y, et al Clinical features of patients infected with 2019 novel coronavirus in Wuhan, China. Lancet. 2020;395(10223):497–506. Epub 2020/01/28. 10.1016/S0140-6736(20)30183-5 . 31986264 PMC7159299

[3] Wang D, Hu B, Hu C, Zhu F, Liu X, Zhang J, et al Clinical Characteristics of 138 Hospitalized Patients With 2019 Novel Coronavirus-Infected Pneumonia in Wuhan, China. JAMA. 2020 Epub 2020/02/08. 10.1001/jama.2020.1585 32031570 PMC7042881

[4] Zhou F, Yu T, Du R, Fan G, Liu Y, Liu Z, et al Clinical course and risk factors for mortality of adult inpatients with COVID-19 in Wuhan, China: a retrospective cohort study. Lancet. 2020 Epub 2020/03/15. 10.1016/S0140-6736(20)30566-3 . 32171076 PMC7270627

[5] Lu S, Zhao Y, Yu W, Yang Y, Gao J, Wang J, et al Comparison of SARS-CoV-2 infections among 3 species of non-human primates. bioRxiv. 2020 Epub 2020/04/08. 10.1101/2020.04.08.031807

[6] Munster VJ, Feldmann F, Williamson BN, van Doremalen N, Perez-Perez L, Schulz J, et al Respiratory disease in rhesus macaques inoculated with SARS-CoV-2. Nature. 2020 Epub 2020/05/13. 10.1038/s41586-020-2324-7 . 32396922 PMC7486227

[7] Shan C, Yao Y, Yang X, Zhou Y, Wu J, Gao G, et al Infection with Novel Coronavirus (SARS-CoV-2) Causes Pneumonia in the *Rhesus Macaques*. ResearchSquare. 2020 10.21203/rs.2.25200/v1 PMC7364749 32636454

[8] Burke RM, Midgley CM, Dratch A, Fenstersheib M, Haupt T, Holshue M, et al Active Monitoring of Persons Exposed to Patients with Confirmed COVID-19—United States, January-February 2020. MMWR Morb Mortal Wkly Rep. 2020;69(9):245–6. Epub 2020/03/07. 10.15585/mmwr.mm6909e1 . 32134909 PMC7367094

[9] Chan JF, Yuan S, Kok KH, To KK, Chu H, Yang J, et al A familial cluster of pneumonia associated with the 2019 novel coronavirus indicating person-to-person transmission: a study of a family cluster. Lancet. 2020;395(10223):514–23. Epub 2020/01/28. 10.1016/S0140-6736(20)30154-9 31986261 PMC7159286

[10] Li Q, Guan X, Wu P, Wang X, Zhou L, Tong Y, et al Early Transmission Dynamics in Wuhan, China, of Novel Coronavirus-Infected Pneumonia. N Engl J Med. 2020;382(13):1199–207. Epub 2020/01/30. 10.1056/NEJMoa2001316 31995857 PMC7121484

[11] Liu J, Liao X, Qian S, Yuan J, Wang F, Liu Y, et al Community Transmission of Severe Acute Respiratory Syndrome Coronavirus 2, Shenzhen, China, 2020. Emerg Infect Dis. 2020;26(6):1320–3. Epub 2020/03/04. 10.3201/eid2606.200239 32125269 PMC7258448

[12] Organization WH. Report of the WHO-China Joint Mission on Coronavirus Disease 2019 (COVID-19) 16–24 February 2020 Geneva: World Health Organization: 2020.

[13] Zhang R, Li Y, Zhang AL, Wang Y, Molina MJ. Identifying airborne transmission as the dominant route for the spread of COVID-19. Proc Natl Acad Sci U S A. 2020. Epub 2020/06/13. 10.1073/pnas.2009637117 . 32527856 PMC7334447

[14] Chen W, Li Z, Yang B, Wang P, Zhou Q, Zhang Z, et al Delayed-Phase Thrombocytopenia in Patients of Coronavirus Disease 2019 (COVID-19). Br J Haematol. 2020 Epub 2020/05/27. 10.1111/bjh.16885 . 32453877 PMC7283673

[15] Finch CL, Crozier I, Lee JH, Byrum R, Cooper TK, Liang J, et al Characteristic and quantifiable COVID-19-like abnormalities in CT- and PET/CT-imaged lungs of SARS-CoV-2-infected crab-eating macaques (*Macaca fascicularis*). bioRxiv 20200514096727. 2020 10.1101/2020.05.14.096727 32511338 PMC7241101

[16] Ephraim GP, Barrows SZ. Clinical Aspects of Primate Medicine. Iowa State University Veterinarian. 1988;50(1):8–13.

[17] Blair RV, Vaccari M, Doyle-Meyers LA, Roy CJ, Russell-Lodrigue K, Fahlberg M, et al ARDS and Cytokine Storm in SARS-CoV-2 Infected Caribbean Vervets. bioRxiv. 2020 Epub 2020/06/18. doi: 10.1101.2020.06.18.157933

[18] Hartman AL, Nambulli S, McMillen CM, White AG, Tilston-Lunel NL, Albe JR, et al SARS-CoV-2 infection of African green monkeys results in mild respiratory disease discernible by PET/CT imaging and prolonged shedding of infectious virus from both respiratory and gastrointestinal tracts. bioRxiv. 2020 Epub 2020/06/20. 10.1101/2020.06.20.137687 PMC7535860 32946524

[19] Borak J. Airborne Transmission of COVID-19. Occup Med (Lond). 2020 Epub 2020/06/02. 10.1093/occmed/kqaa080 . 32476011 PMC7313827

[20] Godri Pollitt KJ, Peccia J, Ko AI, Kaminski N, Dela Cruz CS, Nebert DW, et al COVID-19 vulnerability: the potential impact of genetic susceptibility and airborne transmission. Hum Genomics. 2020;14(1):17 Epub 2020/05/14. 10.1186/s40246-020-00267-3 32398162 PMC7214856

[21] Morawska L, Tang JW, Bahnfleth W, Bluyssen PM, Boerstra A, Buonanno G, et al How can airborne transmission of COVID-19 indoors be minimised? Environ Int. 2020;142:105832 Epub 2020/06/11. 10.1016/j.envint.2020.105832 32521345 PMC7250761

[22] Setti L, Passarini F, De Gennaro G, Barbieri P, Perrone MG, Borelli M, et al Airborne Transmission Route of COVID-19: Why 2 Meters/6 Feet of Inter-Personal Distance Could Not Be Enough. Int J Environ Res Public Health. 2020;17(8). Epub 2020/04/29. 10.3390/ijerph17082932 32340347 PMC7215485

[23] Rockx B, Kuiken T, Herfst S, Bestebroer T, Lamers M, de Meulder D, et al Comparative Pathology of COVID-19, MERS and SARS in a Non-Human Primate Model. bioRxiv. 2020 Epub 2020/03/17. 10.1101/2020.03.17.995639 PMC7164679 32303590

[24] Woolsey C, Borisevich V, Prasad A, Agans K, Deer D, Dobias N, et al Establishment of an African green monkey model for COVID-19. bioRxiv. 2020 Epub 2020/05/17. 10.1101/2020.05.17.100289 33235385 PMC7790436

[25] Corbett KS, Flynn B, Foulds KE, Francica JR, Boyoglu-Barnum S, Werner AP, et al Evaluation of the mRNA-1273 Vaccine against SARS-CoV-2 in Nonhuman Primates. N Engl J Med. 2020;383(16):1544–55. Epub 2020/07/30. 10.1056/NEJMoa2024671 32722908 PMC7449230

[26] Lu S, Zhao Y, Yu W, Yang Y, Gao J, Wang J, et al Comparison of nonhuman primates identified the suitable model for COVID-19. Signal Transduct Target Ther. 2020;5(1):157 Epub 2020/08/21. 10.1038/s41392-020-00269-6 32814760 PMC7434851

[27] Zheng H, Li H, Guo L, Liang Y, Li J, Wang X, et al Virulence and pathogenesis of SARS-CoV-2 infection in rhesus macaques: A nonhuman primate model of COVID-19 progression. PLoS Pathog. 2020;16(11):e1008949 Epub 2020/11/13. 10.1371/journal.ppat.1008949 33180882 PMC7660522

[28] Deng W, Bao L, Liu J, Xiao C, Liu J, Xue J, et al Primary exposure to SARS-CoV-2 protects against reinfection in rhesus macaques. Science. 2020;369(6505):818–23. Epub 2020/07/04. 10.1126/science.abc5343 32616673 PMC7402625

[29] Liu J, Babka AM, Kearney BJ, Radoshitzky SR, Kuhn JH, Zeng X. Molecular detection of SARS-CoV-2 in formalin fixed paraffin embedded specimens. JCI Insight. 2020 Epub 2020/05/08. 10.1172/jci.insight.139042 . 32379723 PMC7406253

[30] Wrapp D, Wang N, Corbett KS, Goldsmith JA, Hsieh CL, Abiona O, et al Cryo-EM structure of the 2019-nCoV spike in the prefusion conformation. Science. 2020;367(6483):1260–3. Epub 2020/02/23. 10.1126/science.abb2507 32075877 PMC7164637

[31] Ricks KM, Shoemaker CJ, Dupuy LC, Flusin O, Voorhees MA, Fulmer AN, et al Development of a bead-based immunoassay using virus-like particles for detection of alphaviral humoral response. J Virol Methods. 2019;270:12–7. Epub 2019/04/19. 10.1016/j.jviromet.2019.04.013 . 30998959

[32] Darriba D, Taboada GL, Doallo R, Posada D. jModelTest 2: more models, new heuristics and parallel computing. Nat Methods. 2012;9(8):772 Epub 2012/08/01. 10.1038/nmeth.2109 22847109 PMC4594756

[33] Guindon S, Gascuel O. A simple, fast, and accurate algorithm to estimate large phylogenies by maximum likelihood. Syst Biol. 2003;52(5):696–704. Epub 2003/10/08. 10.1080/10635150390235520 . 14530136

