# Supplementary figures and images for: Development of a coronavirus disease 2019 nonhuman primate model using airborne exposure
Source: PLoS One. 2021 Feb 2;16(2):e0246366. doi: 10.1371/journal.pone.0246366 (PMC7853502; doi:10.1371/journal.pone.0246366)

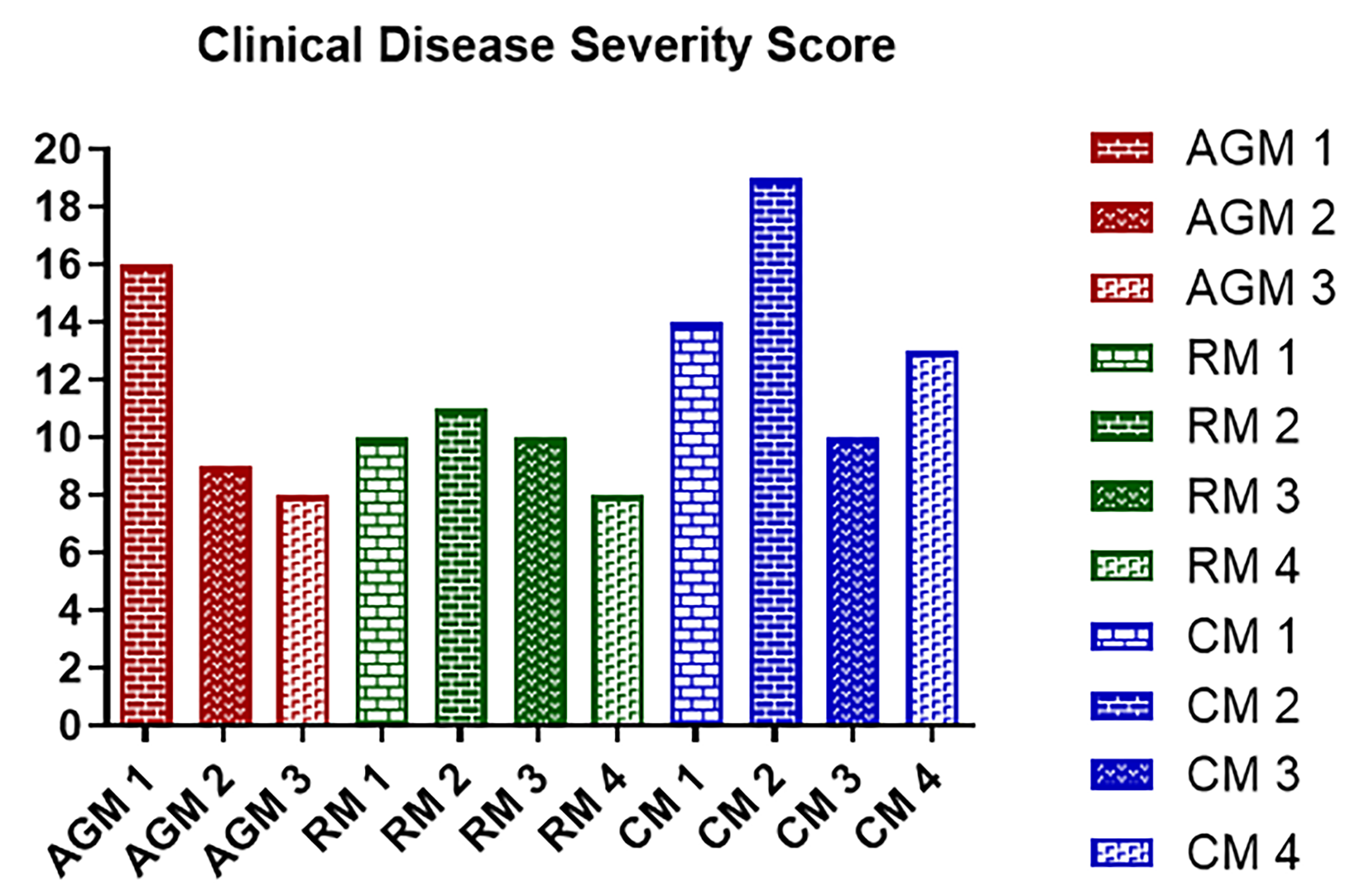

Supplement: S1 Fig — Disease severity based on clinical signs was graded using a scoring system that can be found in S1 Table. (TIF) [file pone.0246366.s001.tif]

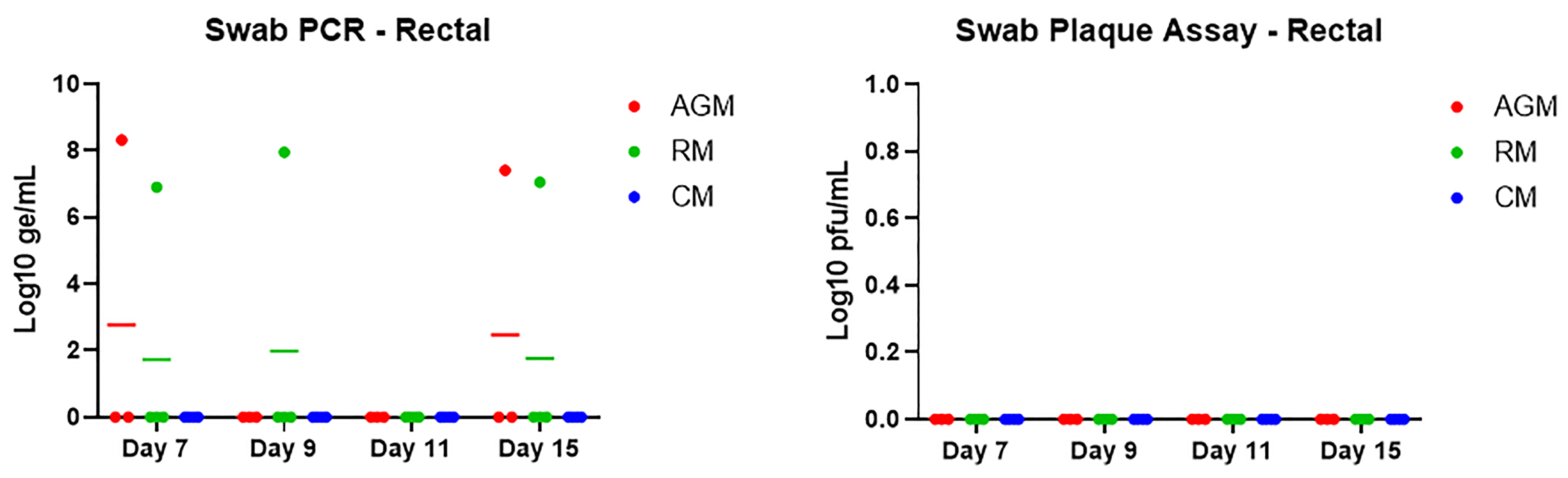

Supplement: S2 Fig — SARS-CoV-2-specific qRT-PCR was performed on RNA extracted from nasopharyngeal and oropharyngeal swab clarified homogenates. Plaque assay was performed on nasopharyngeal and oropharyngeal clarified homogenates. Data are shown as Log10 ge/mL (qRT-PCR) or Log10 pfu/mL (plaque assay). (TIF) [file pone.0246366.s002.tif]

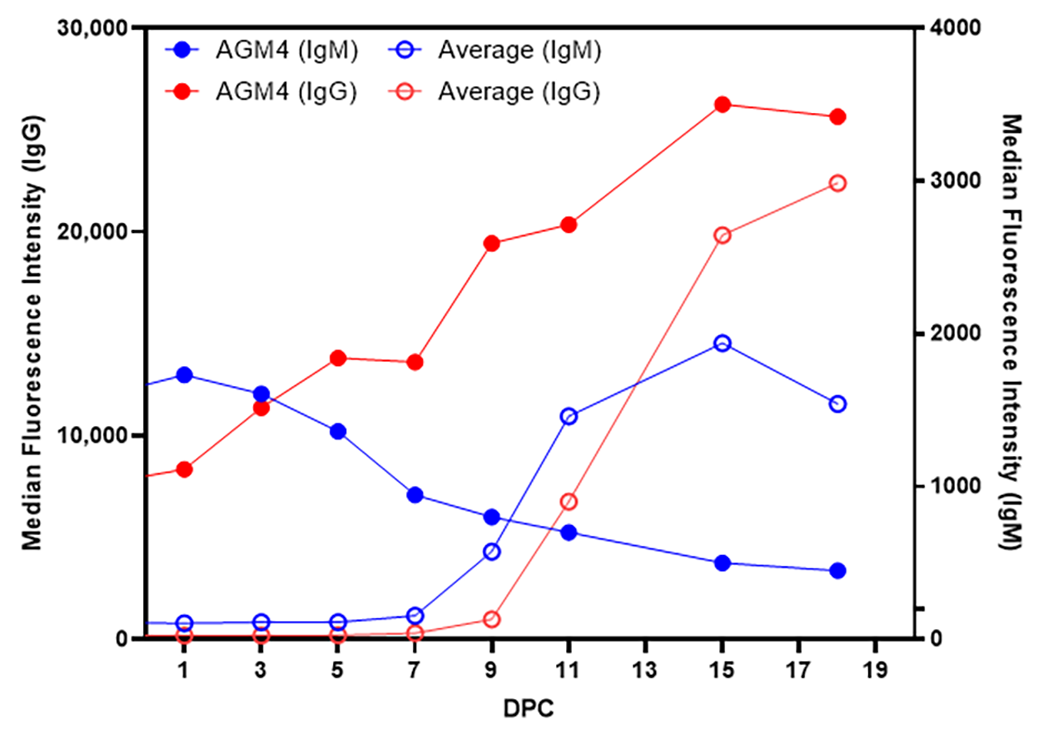

Supplement: S3 Fig — The longitudinal IgM and IgG response against SARS-CoV-2 UT full spike as measured by Magpix multiplex immunoassay is shown. Average = the average of the median fluorescence intensity for the indicated antibody response for CMs, RMs, and AGMs 1–3. (TIF) [file pone.0246366.s003.tif]

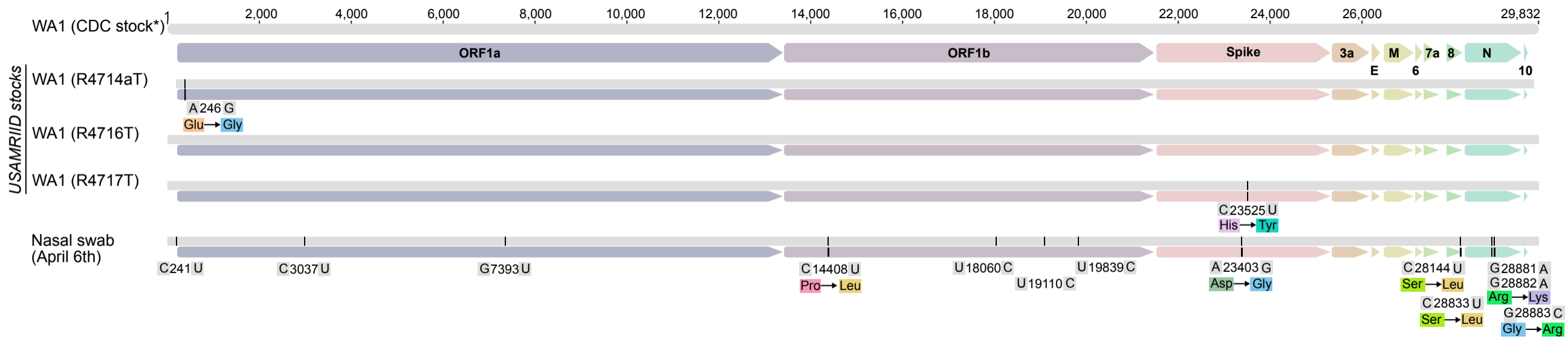

Supplement: S4 Fig — A visual depiction of sequence similarity with highlighted changes between the viral stocks available at USAMRIID and the April 6th sample during the time of the study. (PDF) [file pone.0246366.s004.pdf]

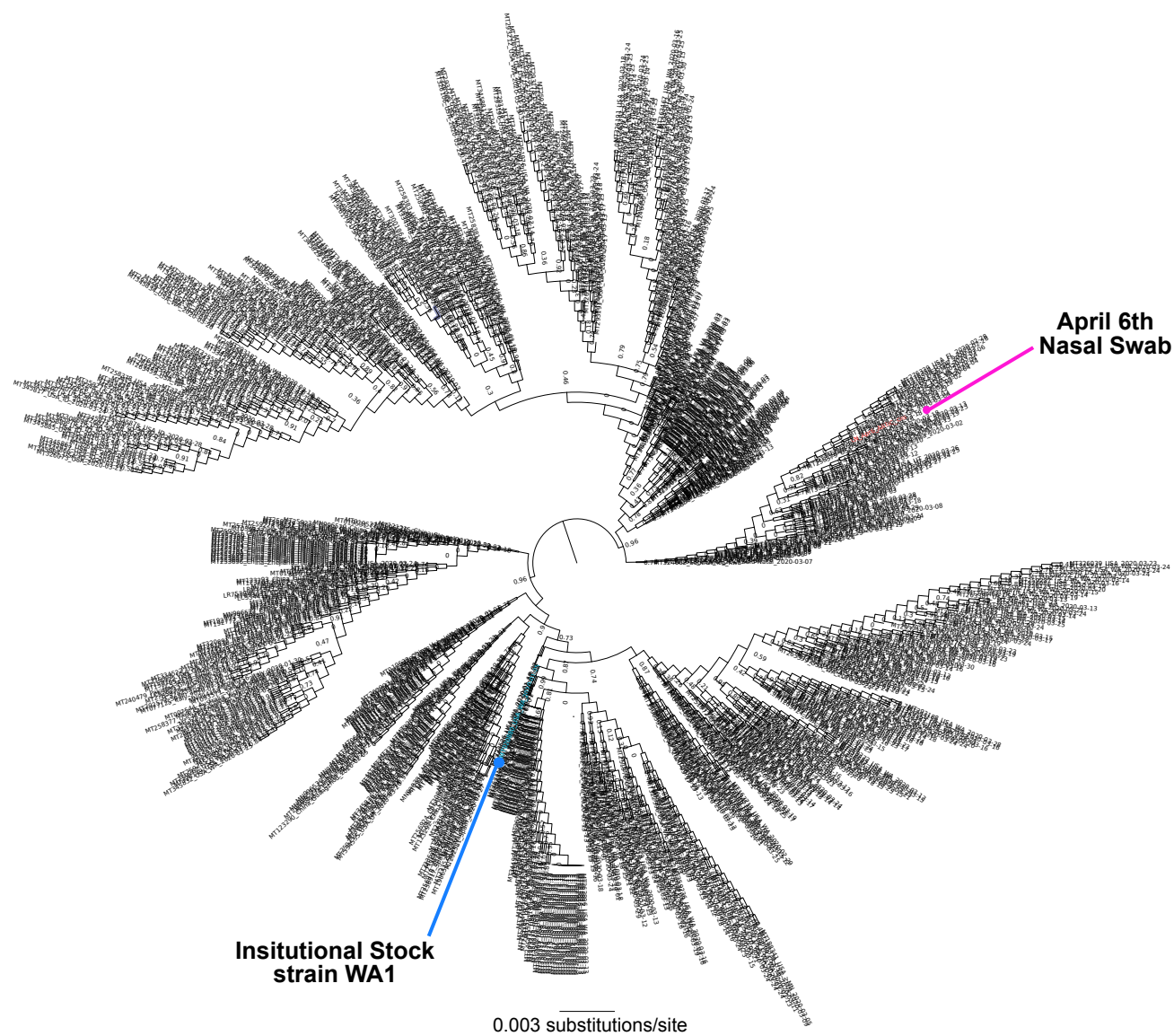

Supplement: S5 Fig — A radial Maximum-likelihood tree estimated using 1,003 SARS-CoV-2 coding-complete genomes. The WA1 strain (Genbank accession number: MT020880) and consensus genome sequence from an infected non-human primate nasal swab collected on April 6th are highlighted using colored circles. Tree branches are scaled by substitutions per site. Tree node support values were generated using 5000 Shimodaira-Hasegawa tests and are shown in decimal form. (PDF) [file pone.0246366.s005.pdf]

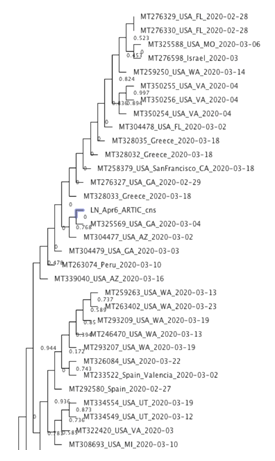

Supplement: S6 Fig — A close view of the near neighbors to the sequence generated April 6th sample. (TIF) [file pone.0246366.s006.tif]
