# Supplementary material for: Development of a coronavirus disease 2019 nonhuman primate model using airborne exposure
Source: PLoS One. 2021 Feb 2;16(2):e0246366. doi: 10.1371/journal.pone.0246366 (PMC7853502; doi:10.1371/journal.pone.0246366)
Supplement: S1 Table — (DOCX) [file pone.0246366.s007.docx]

S1 Table. Nonhuman primate COVID-19 clinical disease severity

| **Parameter** | **Description** | **Score** |
| --- | --- | --- |
| Responsiveness | Alert, responsive, normal species specific behavior | 0 |
|  | Slightly diminished general activity, subdued but responds normally to external stimuli | 1 |
|  | Withdrawn, may have head down, upright fetal posture, hunched, reduced response to external stimuli | 2 |
|  | Prostrate but able to rise if stimulated, or dramatically reduced response to external stimuli | 6 |
|  | Persistently prostrate (unable to rise when stimulated), or severely or completely unresponsive, | 8 |
| Discharge | Nasal or ocular | 2 |
|  | Nasal and ocular | 4 |
| Integument | Rash or unusual bruising | 2 |
|  | Rash and unusual bruising | 4 |
| Respiratory Function | Normal - no apparent changes in breathing, 30-50 breaths per minute, and no cough | 0 |
|  | Mild dysfunction - 50-65 breaths per minute, increased respiratory sounds on auscultation, or isolated cough | 2 |
|  | Moderate dysfunction - increased effort of breathing (abdominal breathing and/or nasal flare), 66-80 breaths per minute, or apparent cough | 4 |
|  | Severe dysfunction – continuous gasping, open mouth breathing + abdominal breathing, and/or cyanosis | 8 |
| Food consumption | Evidence of biscuit and fruit consumption | 0 |
|  | No evidence of biscuit or fruit consumption | 1 |
|  | No evidence of biscuit and fruit consumption | 4 |
| Stool/GI | Normal | 0 |
|  | Soft or liquid stool present, palpable gas or palpable fluid in GI region | 1 |
|  | Rectal bleeding | 2 |
| Body Temperature | Normal | 0 |
|  | Temperature elevated (>3 standard deviations above baseline) | 2 |
|  | Fever (>1.5°C above baseline) | 4 |
| **Parameter** | **Description** | **Score** |
| Heart rate | Normal (up to 19 BPM over baseline) | 0 |
|  | Mild tachycardia (20-39 BPM over baseline) | 1 |
|  | Moderate tachycardia (40-69 BPM over baseline) | 2 |
|  | Severe tachycardia (>70 BPM over baseline) | 3 |
| Respiratory rate | Normal - 30-50 breaths per minute | 0 |
|  | Mild tachypnea- 50-65 breaths per minute | 2 |
|  | Moderate tachypnea - 66-80 breaths per minute | 4 |
|  | Severe tachypnea - >80 breaths per minute | 6 |
| SpO_2_ | Normal (95-100%) | 0 |
|  | Mildly decreased (90-94%) | 2 |
|  | Moderately decreased (87-89%) | 4 |
|  | Severely decreased (<87%) | 6 |
| Body weight | Normal (0-3% loss) | 0 |
|  | Mild (4-9% loss) | 1 |
|  | Moderate (10-16% loss) | 2 |
|  | Severe (>16% loss) | 3 |
| Clotting following blood collection | Normal – site of venipuncture clots quickly | 0 |
|  | Abnormal – noticeable increase in time required for the animal to clot following venipuncture | 2 |
| Gait, locomotion, and balance | Normal | 0 |
|  | Mild – shaking | 1 |
|  | Moderate – difficulty grasping items, or difficulty moving around cage | 4 |
|  | Severe – unable to moving around cage, unable to climb, or falling down | 6 |
| Lymphadenopathy | Absent | 0 |
|  | Present | 1 |

| **Parameter** | **Description** | **Score** |
| --- | --- | --- |
| Conjunctival Erythema | Absent | 0 |
|  | Present | 1 |
| Radiographic Findings | Normal | 0 |
|  | Mild – opacity/glassy appearance or infiltrates in at least one lung lobe | 1 |
|  | Moderate – opacity/glassy appearance or infiltrates in at least two lung lobes | 2 |
|  | Severe – opacity/glassy appearance or infiltrates in at least three lung lobes | 3 |
