# Supplementary material for: Development of a coronavirus disease 2019 nonhuman primate model using airborne exposure
Source: PLoS One. 2021 Feb 2;16(2):e0246366. doi: 10.1371/journal.pone.0246366 (PMC7853502; doi:10.1371/journal.pone.0246366)
Supplement: S2 Table — (DOCX) [file pone.0246366.s008.docx]

**S2 Table. Summary clinical pathology findings**

| Animal Number | AGM 1 | AGM 2 | AGM 3 | RM 1 | RM 2 | RM 3 | RM 4 | CM 1 | CM 2 | CM 3 | CM 4 |
| --- | --- | --- | --- | --- | --- | --- | --- | --- | --- | --- | --- |
| WBC  (+/-) | X  (-39%)  (+30%) | X  (-37%) | X (+69%) | X  (-28%) | X  (+36%) | X  (-29%)  (+31%) | X (+28) | X  (-29%) | X  (-44%) | X  (-52%) | X  (-29%) |
| NEU  (+/-) | X  (-58%) | X  (-56%) | X  (-55%)  (+80%) | X  (-30%) | X  (+44%)  (-34%) | X  (-58%)  (+58%) | X  (-46%) | X  (-31%) | X  (-59%) | X  (-60%) | X  (-34%) |
| LYM  (+/-) | X  (-31%)  (+52%) | X  (-99%)  (+52%) | X  (-95%)  (+155%) | X  (-27%) | X  (-29%)  (+33%) | X  (-68%)  (+32%) | X  (-41%)  (+79%) | X  (-54%)  (+96%) | X  (-43%)  (+108%) | X  (+90%) | X  (-72%) |
| MON  (+/-) | X  (-33%)  (+167%) | X  (+5,633%) | X  (+4,300%) | X  (+80%) | X  (+167%) | X  (+67%)  (-50%) | X  (+125%)  (-25%) | X  (+220%) | X  (+314%) | X  (-50%)  (+33%) | X  (-55%)  (+64%) |
| PLT  (+/-) | X  (-51%) | X  (-43%) | X  (-52%) | X  (-42%) |  |  |  |  |  |  | X  (-38%) |
| ALT  (+) | X  (+84%) | X  (+36%) |  | X  (+64%) |  | X  (+42%) | X  (+48%) | X  (+76%) | X  (+85%) | X  (+34%) | X  (+48%) |
| ALB  (-) |  |  |  |  |  |  |  |  | X  (-39%) |  |  |
| ALP  (+) | X  (+32%) |  | X  (+37%) |  |  |  |  | X  (+165%) |  |  | X  (+35%) |
| AST  (+) | X  (+127%) | X  (+117%) | X  (+29%) | X  (+29%) | X  (+56%) |  | X  (+49%) | X  (+153%) | X  (+46%) | X  (+79%) | X  (+126%) |
| GGT  (+) | X  (+40%) | X  (+30%) | X  (+57%) |  |  |  |  | X  (+129%) |  |  |  |
| GLU  (+/-) | X  (+63%) | X  (+69%) | X  (+41%) |  |  |  |  |  | X  (+33%) | X (+26%) | X  (+45%) |

X = >25% change from baseline for a given parameter for at least 1 time point

() = maximum percent change from baseline noted for an animal
